# Phytochemical Profiling of Mulberry Diels-Alder Adducts as Selective Butyrylcholinesterase Inhibitors: In Vitro Activity, Molecular Docking, and Molecular Dynamics Simulation

**DOI:** 10.3390/molecules31101574

**Published:** 2026-05-08

**Authors:** Xiang Cui, Xiu-Cheng Zhu, Shu-Qi Yao, Rui Wang, Yun-Xia Zhang, Jin Li, Biao Wang, Yan-Ru Deng, Chang-Jing Wu

**Affiliations:** 1 College of Life Sciences and Agronomy, Zhoukou Normal University, Zhoukou 466001, China; 2 Fuxi Laboratory, Zhoukou Normal University, Zhoukou 466001, China; 3 College of Traditional Chinese Medicine, Tianjin University of Traditional Chinese Medicine, Tianjin 301617, China; 4 Field Observation and Research Station of Green Agriculture in Dancheng County, Zhoukou 466001, China

**Keywords:** Cortex Mori Radicis, Diels–Alder adducts, butyrylcholinesterase, Alzheimer’s disease, enzyme inhibition, molecular docking, natural products

## Abstract

Alzheimer’s disease (AD) is a common neurodegenerative disorder linked to cholinergic dysfunction, with butyrylcholinesterase (BChE) being a key therapeutic target for moderate–severe AD. Cortex Mori Radicis, a traditional Chinese medicinal herb, is rich in Diels–Alder adducts with potential neuroprotective effects; here, eighteen Diels–Alder adducts (four new: morusalbanol B–E, **1**–**4**) were isolated and identified from its 80% ethanol extract. Their cholinesterase inhibitory activities were assessed via Ellman’s method, with enzyme kinetics and molecular docking performed for active compounds. Most compounds showed selective BChE inhibition, with kuwanon X (**14**) being the most potent (IC_50_ = 2.3 μM). morusalbanol B (**1**), cathayanon A (**8**), and kuwanon G (**12**) acted as noncompetitive inhibitors, while Morusalbanol C (**2**) and kuwanon X (**14**) were mixed competitive inhibitors. Molecular docking suggested that potent inhibitors occupied the BChE active pocket via hydrogen bonds, π-π stacking, and hydrophobic interactions with Trp82, His438, and Phe329. MD simulations and MM-GBSA binding free energy analysis further verified that all three representative complexes (**1**, **8**, and **14**) achieved favorable thermodynamic and structural stability, with binding driven primarily by van der Waals forces. Residue decomposition revealed that Trp82 and Phe329 served as core binding hotspots for all tested inhibitors. Structure–activity analysis indicated that a cis-trans methylcyclohexene configuration, shorter aliphatic ester chains, and more prenyl groups enhanced BChE inhibition. This study provides new lead compounds and a systematic molecular mechanism basis for developing novel anti-AD BChE inhibitors from natural products.

## 1. Introduction

Alzheimer’s disease (AD) is the most common fatal neurodegenerative brain disorder, characterized by progressive cognitive and behavioral deficits. With the intensification of global population aging, the number of AD patients is growing continuously, which has become a major public health issue seriously threatening the health of the elderly. It is estimated that the global population of AD patients will rise from 50 million in 2015 to 132 million by 2050 [1]. Multiple hypotheses explain its pathogenesis, including amyloid-β deposition, tau protein aggregation, neuroinflammation, and cholinergic dysfunction, with the latter being a well-recognized therapeutic target [2]. Cholinergic neuron damage in AD leads to reduced acetylcholine (ACh) levels, impairing learning and memory; inhibiting cholinesterase (ChE) is an effective way to elevate brain ACh. Two key ChEs exist: acetylcholinesterase (AChE), the main target of clinical drugs like donepezil and galantamine, and butyrylcholinesterase (BChE), which becomes the primary ACh-metabolizing enzyme in moderate-to-severe AD, making it a promising specific target [3]. In recent decades, natural products (NPs) have emerged as important sources for novel BChE inhibitors, with many NP derivatives showing excellent anti-AD activity in animal models [4,5,6,7].

Cortex Mori Radicis, the dried root bark of *Morus alba* L., is a traditional Chinese medicinal herb with efficacies of clearing lung heat, relieving asthma, and inducing diuresis. Modern pharmacology has confirmed its anti-inflammatory, antioxidant, antibacterial, and prominent neuroprotective effects, suggesting potential in AD treatment [8]. Its main bioactive constituents include Diels–Alder-type adducts, named mulberry Diels–Alder-type adducts (MDAAs), with over 160 structurally characterized to date, divided into eight subclasses based on dienophile moieties [9]. As multi-target NPs, MDAAs exhibit diverse activities, including anti-tumor, anti-neurodegenerative, and enzyme inhibitory effects; for example, kuwanon G potently inhibits AChE, BChE, and BACE1, key enzymes linked to AD [10]. Previous studies have identified BChE-inhibitory constituents like moracin derivatives and arylbenzofurans from Cortex Mori Radicis, proving it a valuable source of anti-AD NPs [11,12]. However, systematic investigations of MDAAs from this herb, their structure–activity relationships (SARs), and the BChE binding mechanisms remain insufficiently explored. This study systematically isolated eighteen Diels–Alder adducts (four new ones) from Cortex Mori Radicis, evaluated their selective BChE-inhibitory activity, clarified their inhibition kinetics, and explored the binding mechanisms using molecular docking, molecular dynamics simulation, and MM-GBSA binding free energy decomposition. The work provides novel natural lead compounds and a dynamic molecular basis for the development of anti-AD selective BChE inhibitors, filling the gap in systematic research on MDAAs as BChE inhibitors.

## 2. Results and Discussion

### 2.1. Structure Determination of Compounds **1**–**18**

**Figure 1:**
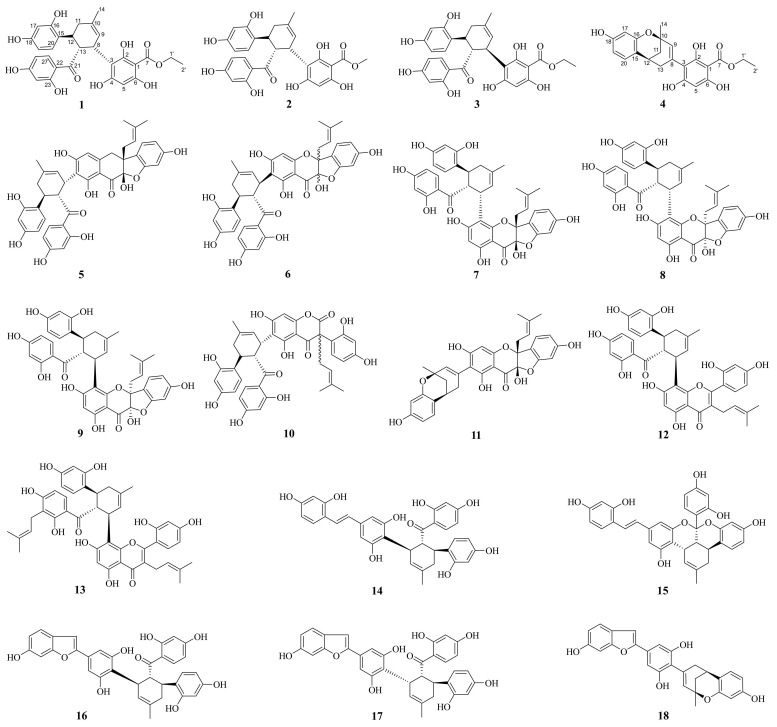
Structures of compounds **1**–**18** isolated from Cortex Mori Radicis.

Eighteen Diels–Alder adducts were isolated from the 80% ethanol-eluted partition on AB-8 macroporous resin column chromatography of Cortex Mori Radicis ethanolic extract. Based on the comprehensive analysis of various spectroscopic data—including high-resolution electrospray ionization mass spectra (HR-ESI-MS), 1D/2D nuclear magnetic resonance (NMR) spectra, and ultraviolet (UV) spectra—four previously undescribed compounds were elucidated and named morusalbanol B–E (**1**–**4**), and fourteen known compounds were identified to be sanggenon C (**5**) [13], sanggenon D, (**6**) [14], sanggenon O (**7**) [13], cathayanon A (**8**) [13], cathayanon B (**9**) [13], sanggenon Q (**10**) [15], sanggenon B (**11**) [16], kuwanon G (**12**) [17], kuwanon H (**13**) [18], kuwanon X (**14**) [19], kuwanol A (**15**) [20], mulberrofuran J (**16**) [19], mulberrofuran C (**17**) [21], and mulberrofuran H (**18**) [22] by comparison with the reported NMR data (Figure 1).

**Figure 2:**
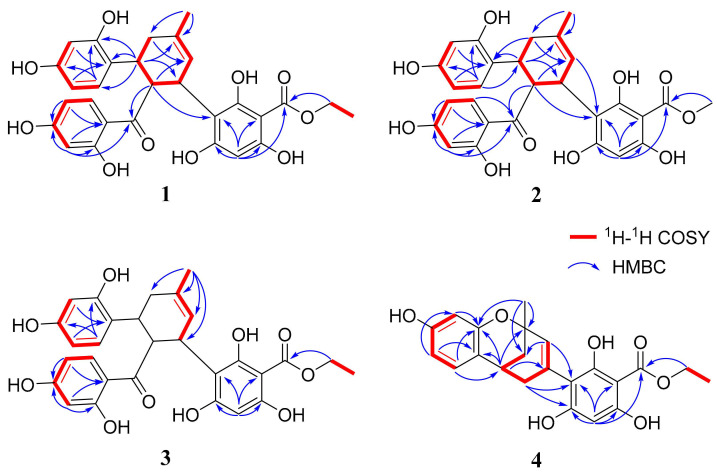
Key ^1^H-^1^H COSY and HMBC correlations of compounds **1**–**4**.

**Figure 3:**
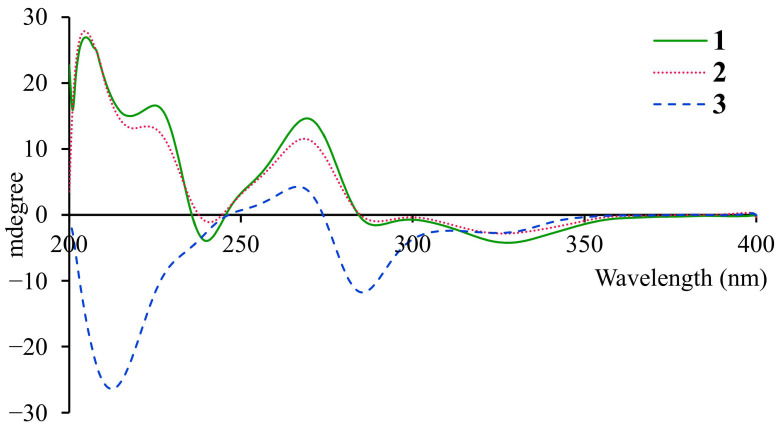
ECD spectra of compounds **1**–**3**.

Compound **1** was obtained as a pale yellow amorphous powder with a positive specific optical rotation ([α]) value of +158.3 (MeOH). Its UV spectrum exhibited characteristic maximum absorption bands at 220, 275, and 320 nm. The high-resolution electrospray ionization mass spectrometry (HR-ESI-MS) displayed a quasi-molecular ion peak at *m*/*z* 537.1768 [M+H]^+^, which was in good agreement with the calculated value of 537.1761 for C_29_H_29_O_10_, thus establishing the molecular formula as C_29_H_28_O_10_ with an index of hydrogen deficiency (IHD) of 16. The ^1^H NMR spectrum (Table 1) featured two sets of ABX-type aromatic proton signals at δ_H_ 8.16 (1H, d, J = 8.9 Hz, H-27), 6.26 (1H, dd, J = 8.9, 2.3 Hz, H-26), and 6.13 (1H, d, J = 2.3 Hz, H-24) and δ_H_ 6.88 (1H, d, J = 8.4 Hz, H-20), 6.30 (1H, d, J = 2.4 Hz, H-17), and 6.19 (1H, dd, J = 8.4, 2.5 Hz, H-19), along with a singlet aromatic proton at δ_H_ 5.78 (1H, s, H-5). Signals attributable to a trisubstituted methylcyclohexene moiety were observed at δ_H_ 4.11 (1H, d, J = 6.4 Hz, H-8), 5.52 (1H, br s, H-9), 2.46 (1H, dd, J = 17.7, 4.8 Hz, Hα-11), 2.20 (1H, br d, J = 17.2 Hz, Hβ-11), 3.77 (1H, q, J = 5.0 Hz, H-12), 4.45 (1H, t, J = 5.0 Hz, H-13), and 1.86 (3H, br s, H-14). Additionally, an oxygen-bearing ethyl group was identified by the proton signals at δ_H_ 4.44 (2H, q, J = 7.0 Hz, H-1′) and 1.37 (3H, t, J = 7.0 Hz, H-2′). The proton–proton coupling correlations were confirmed by the ^1^H-^1^H COSY experiment (Figure 2), and the corresponding proton–carbon connectivities were established via the HSQC experiment. The ^13^C NMR spectrum of compound **1** revealed a total of 29 carbon resonances (Table 1). Besides the carbons correlated with the aforementioned protons, 14 quaternary carbon signals were assigned, including a ketone carbonyl at δ_C_ 209.6 (C-21), an ester carbonyl at δ_C_ 171.3 (C-7), seven oxygenated *sp*^2^-hybridized carbons at δ_C_ 156.9 (C-16), 157.6 (C-18), 161.8 (C-4), 162.3 (C-2), 164.5 (C-6), 166.3 (C-25), and 166.7 (C-23), as well as five unoxygenated *sp*^2^-hybridized carbons at δ_C_ 94.2 (C-1), 108.5 (C-3), 114.5 (C-22), 123.4 (C-15), and 134.4 (C-10). The HMBC experiment of **1** (Figure 2) further verified the presence of the methylcyclohexene moiety by the significant correlations of H-14 with C-9, C-10, and C-11, and H-11 with C-9 and C-10. Strong HMBC correlations of H-5 with C-1, C-3, C-4, and C-6, together with weak correlations with C-2 and C-7, and the correlation of H-1′ with C-7, indicated the existence of an ethyl 2,4,6-trihydroxybenzoate moiety in the molecule. Moreover, the HMBC correlations of H-17 and H-19 with C-15, H-20 with C-16 and C-18, H-24 with C-22, C-23, and C-25, and H-26 with C-22 and C-25 allowed the unambiguous assignment of all proton and carbon signals for the two 2,4-dihydroxytrisubstituted benzene moieties. Subsequent analysis of the HMBC correlations was employed to connect the aforementioned structural fragments. Firstly, the correlations of H-12 with C-15, C-16, and C-20 linked one trisubstituted benzene moiety to C-12 of the methylcyclohexene ring. Secondly, a strong HMBC correlation of H-13 with C-21 and a weak correlation of H-24 with C-21 suggested that the other trisubstituted benzene moiety was attached to C-13 of the methylcyclohexene ring via the carbonyl carbon C-21. Furthermore, a strong HMBC correlation of H-13 with C-3 confirmed the covalent linkage between the ethyl 2,4,6-trihydroxybenzoate moiety and the methylcyclohexene moiety through a C-3–C-8 bond. On the basis of the above evidence, the planar structure of compound **1** was elucidated as a Diels–Alder adduct, as depicted in the figure above. Morus plants are rich in Diels–Alder adducts, which are classified into two major types (cis-trans and all-trans) based on the relative configurations of the substituents on the methylcyclohexene ring. In compound **1**, the coupling constants of H-8/H-13 and H-12/H-13 were both 5.0 Hz, indicating a cis-trans relative configuration for C-8, C-12, and C-13. Compound **1** exhibited positive optical activity, and its CD spectrum showed a predominantly positive Cotton effect (Figure 3). According to the exciton chirality method, the absolute configuration of compound **1** was determined as 8*S*, 12*S*, 13*R* [23]. To the best of our knowledge, this compound has not been reported in the literature previously, and thus it was designated as morusalbanol B.

Compound **2** was obtained as a pale yellow amorphous powder, showing a positive optical rotation of [α]_D_ +176.5 (MeOH). Its UV spectrum exhibited maximum absorption peaks at 220, 275, and 320 nm. The HR-ESI-MS displayed a quasi-molecular ion peak at *m*/*z* 521.1448 [M−H]^-^, which was consistent with the calculated value of 521.1448 for C_28_H_25_O_10_, thus establishing its molecular formula as C_28_H_26_O_10_, which is 14 daltons less than that of compound **1**. The ^1^H NMR and ^13^C NMR spectra of compound **2** were extremely similar to those of **1**; however, compared with **1**, **2** lacked the signals of one oxygen-bearing ethyl group and exhibited an additional oxygen-bearing methyl group, as indicated by the proton signal at δ_H_ 3.91 (3H, s, H-1′) and the carbon signal at δ_C_ 52.7 (C-1′) (Table 2). Comprehensive analysis of its 1D and 2D NMR data confirmed the planar structure of compound 2, as depicted in Figure 1. In compound **2**, the coupling constants of H-8/H-13 and H-12/H-13 were both 5.5 Hz, which also identified it as a cis-trans Diels–Alder adduct. The CD spectrum of compound **2** was basically consistent with that of **1** (Figure 3); therefore, it was determined to have the same absolute configuration as **1**, namely 8*S*, 12*S*, 13*R*, and was designated as morusalbanol C.

Compound **3** was obtained as a pale yellow amorphous powder with a negative optical rotation of [α]_D_ −136.1 (MeOH). Its UV absorption characteristics were similar to those of compounds **1** and **2**. Based on the quasi-molecular ion peak at *m*/*z* 535.1605 [M−H]^−^ provided by HRESIMS, which was consistent with the calculated value of 535.1604 for C_29_H_27_O_10_, the molecular formula was established as C_29_H_28_O_10_, which is the same as that of **1**. Comprehensive analysis of the 1D and 2D NMR data of compound **3** revealed that its ^1^H NMR and ^13^C NMR spectra exhibited two sets of signals at room temperature, with significant differences between many pairs of signals. Through analysis, it could be determined that the molecular structure contained two 1,2,4-trisubstituted benzene rings and an ethyl 2,4,6-trihydroxybenzoate structural fragment identical to those in compound **1**, suggesting that it is a stereoisomer of **1**, i.e., an all-trans Diels–Alder adduct. For this type of compound, the cyclohexene ring exists in two conformations in solution with a large energy difference, leading to a slow flipping rate. During the NMR measurement, the carbon and hydrogen signals of the two conformations and their intermediate states may be recorded at room temperature, usually resulting in “broad and fat” shapes of the carbon and hydrogen signals on the methylcyclohexene ring, or even undetectable signals [24]. In the room-temperature NMR spectrum of **3**, the signals at δ_H_ 4.34/4.27 (1H, br d, J = 8.2 Hz, H-8) and 2.13 (br d, J = 14.7 Hz, Hβ-11) appeared as broad peaks with relevant correlations in the 2D spectrum. The signals at δ_H_ 2.52 (1H, br s, Hα-11) and 3.57 (1H, br s, H-12) showed “broad and fat” shapes without relevant correlations in the 2D spectrum, while H-13 was not observed in either the 1D or 2D NMR spectra. In addition, under conformational isomerism, the groups outside the cyclohexene ring are in different chemical environments, especially the shielding or deshielding effects brought by each benzene ring, resulting in paired signals for many hydrogen atoms, including δ_H_ 6.05/5.99 (1H, br d, J = 8.3 Hz, H-19), 5.83/5.66 (1H, s, H-5), 4.52/4.41 (1H, q, J = 7.0 Hz, H-1′), and 1.44/1.36 (3H, t, J = 7.0 Hz, H-2′), etc.; many carbon atoms also exhibited paired signals: δ_C_ 94.2/93.5 (C-1), 162.8/161.3 (C-2), 161.8/161.6 (C-4), 96.6/95.5 (C-5), 165.1/163.6 (C-6), 171.2/171.4 (C-7), 39.0/38.7 (C-8), 126.0/125.8 (C-9), 108.1/107.9 (C-26), and 14.7/14.5 (C-2′). Compound **3** exhibited negative optical activity, and its CD spectrum mainly showed a negative Cotton effect (Figure 3), which was opposite to those of compounds **1** and **2**, consistent with the chirality rule of all-trans Diels–Alder adducts [23]. Based on the above comprehensive analysis, the structure of compound **3** was determined as shown in Figure 1, with the absolute configuration of 8*R*, 12*S*, 13*R*, and was designated as morusalbanol D.

**Figure 4:**
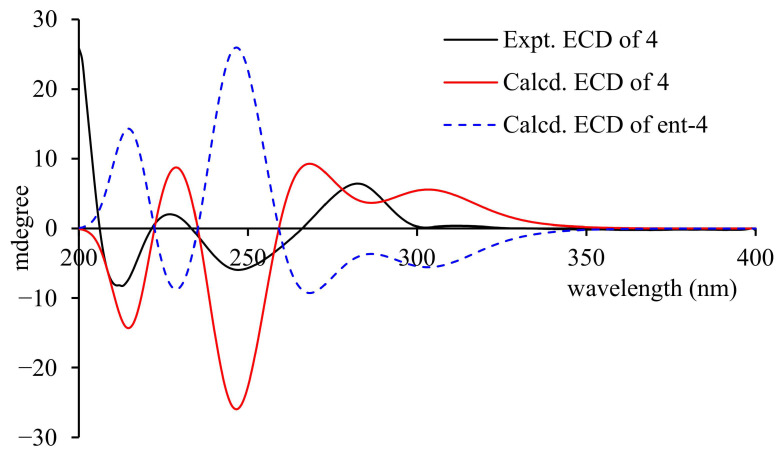
The experimental and calculated ECD spectra of compound **4**.

Compound **4** was obtained as a pale yellow solid. The high-resolution ESI-MS displayed a quasi-molecular ion peak at *m*/*z* 399.1437 [M+H]^+^, suggesting its molecular formula as C_22_H_22_O_7_ with an IHD of 12. The carbon and hydrogen signals in the compound were assigned by analyzing the ^1^H-NMR, ^13^C-NMR, HSQC, COSY, and HMBC spectra. The ^13^C-NMR spectrum showed 22 carbon resonances, including one ester carbonyl carbon, five oxygenated sp^2^-hybridized carbons, nine sp^2^-hybridized carbons, two oxygenated sp^3^-hybridized carbons, and five sp^3^-hybridized carbons. Based on the characteristics of carbon signals and the requirement of IHD, it was speculated that the molecule contained two benzene rings. According to the ABX coupling system of the proton signals at δ_H_ 6.88 (1H, d, J = 8.2 Hz, H-20), 6.29 (1H, dd, J = 8.2, 2.4 Hz, H-19), and 6.17 (1H, d, J = 2.4 Hz, H-17), one of the benzene rings was speculated to be a 1,3,5-trisubstituted benzene ring. The long-range proton–carbon correlations between δ_H_ 6.88 (H-20) and δ_C_ 155.8 (C-16), and between δ_H_ 6.29 (H-19) and δ_C_ 120.0 (C-15) in the HMBC spectrum, confirmed the correctness of the above speculation. In addition, the relevant signals in the HSQC spectrum also indicated the presence of three sp^3^ methylene groups, one sp^3^-hybridized methine group, two sp^2^-hybridized methine groups, and two methyl groups in the molecule. Combined with the ^1^H-^1^H COSY signals, it was speculated that there were an oxygen-bearing ethyl group and a methylcyclohexene moiety, the latter of which was also confirmed by the relevant HMBC signals (Figure 2). The remaining one sp^2^-hybridized methine group was speculated to belong to a pentasubstituted benzene ring. Combined with the characteristics of carbon signals, this benzene ring was speculated to be a 1,3,5-trihydroxy-substituted benzoic acid fragment. The HMBC correlation between δ_H_ 4.45 (H-1′) and δ_C_ 171.25 (C-7) indicated that the aforementioned oxygen-bearing ethyl group formed ethyl benzoate. In the HMBC spectrum, the correlations between δ_H_ 6.88 (H-20) and δ_C_ 32.46 (C-12), and between δ_H_ 3.07 (H-12), δ_C_ 120.0 (C-15), and δ_C_ 155.8 (C-16), indicated that the methylcyclohexene ring was connected to the trisubstituted benzene ring via C-12 and C-15. The HMBC correlations between H-9, H-13, and C-3 suggested that the methylcyclohexene ring was connected to the pentasubstituted benzene ring via C-8 and C-3. At this point, there was still a requirement for 1 IHD in the molecule. The downfield signal of δ_C_ 72.5 (C-10) indicated that it was connected to an oxygen atom, leading to further ring formation. In the HMBC spectrum of **4**, there was an obvious weak correlation signal between H-14 and C-16, suggesting that C-10 was connected to C-16 of the trisubstituted benzene ring via an oxygen atom. Thus, the planar structure of the compound was determined, which was a product of compound **1** and/or **3** with the benzoyl fragment removed and further ring formation. This type of structure is relatively rare, and only four compounds have been reported so far, namely sanggenon B (**11**), mulberrofuran H (**18**), sanggenon S, and sanggenon R. Restricted by the rigid structure of the benzene ring, C-15 and 16-O can only be located on the same side of the cyclohexene ring. The specific rotation of **4** was +36°, which had the same optical activity as mulberrofuran H (**18**) [22]. Combined with the biosynthetic mechanism, the absolute configuration of **4** was preliminarily speculated to be 10*R*, 12*R*. Furthermore, we measured and calculated the ECD spectrum of **4**. The structure of **4** was optimized using DFT theory, and four dominant conformations were obtained using the MMFF94s force field (Figure S1); the four conformations were subjected to further optimizations and ECD calculation, respectively. All conformational ECD spectra were fitted and compared with the experimental ECD spectrum, and it was found that the experimental ECD spectrum was basically consistent with the calculated ECD spectrum of the 10*R*, 12*R* configuration (Figure 4). In summary, the structure of compound **4** was determined as shown in Figure 1, with the absolute configuration of 10*R*, 12*R*, and was designated as morusalbanol E.

### 2.2. ChE Inhibitory Activities

The cholinesterase inhibitory activities of Diels–Alder adducts (**1**–**18**) were screened, with the results summarized in Table 2. The novel Diels–Alder adducts morusalbanol B–D (**1**–**3**) exhibited favorable inhibitory activities. In comparison, compound **1** (ethyl ester, IC_50_ = 6.5 μM)) showed stronger BChE inhibition than **2** (methyl ester, IC_50_ = 19.6 μM). A longer aliphatic ester chain enhances hydrophobic interaction with the BChE active pocket, and improves inhibitory activity. In contrast, **3** displayed a significant reduction in activity compared with **1**, indicating that the three-dimensional structure of the cis-trans Diels–Alder adduct has a higher degree of fit with the BChE binding site. Compounds **1**–**3** exhibited no significant inhibitory activity against AChE (IC_50_ > 100 μM), which endows them with potential value as selective BChE inhibitors for the treatment of moderate-to-severe AD. As the debenzoyl derivative of **1**/**3**, compound **4** exhibited no inhibitory activity against either AChE or BChE, which indicates the crucial role of the benzoyl group in conferring BChE-inhibitory activity to MDAAs. Compounds **5**–**9** are Diels–Alder adducts formed by the conjugation of sanggenon A-type dihydroflavones with chalcones, all of which exerted potent inhibitory effects on BChE while showing remarkable differences in their actions toward AChE. Specifically, compounds with the cyclohexene ring linked at the C-6 position (**5** and **6**) had no obvious inhibitory effect on AChE, whereas C-8-substituted analogs (**7**–**9**) exhibited moderate AChE inhibition. Sanggenon Q (**10**) is structurally derived from the addition of dihydroisoflavones to chalcones followed by prenylation at the C-3 position, and it may also be formed via the isomerization of sanggenon C (**5**); it displayed BChE-inhibitory activity comparable to that of **5**. Sanggenon B (**11**) is a derivative of **5** through debenzoylation and subsequent cyclization, and its BChE-inhibitory activity was only half that of **5**, demonstrating that the chalcone-derived benzoyl group contributes to the biological activity to a certain extent.

Kuwanon G (**12**) is a Diels–Alder adduct of prenylated flavones and chalcone derivatives. As the most abundant MDAA in Cortex Mori Radicis [24], kuwanon G (**12**) exhibits diverse biological functions: it can dose-dependently downregulate the expression of inflammatory factors and NF-κB in LPS-induced RAW264.7 cells [25]; prevent the pathological progression of allergic asthma by inhibiting pulmonary inflammatory and immune damage [26]; exert anti-allergic and anti-inflammatory effects by blocking related signaling pathways and inhibiting enzyme activation [27]; it has obvious hypotensive effect [17]; and can alleviate atherosclerosis by regulating lipid metabolism and inhibiting NF-κB activity [28]. Moreover, kuwanon G has been reported to potently inhibit AChE, BChE, and BACE1—three key enzymes associated with AD pathogenesis—with IC_50_ values of 37.07, 15.3, and 1.01 μM, respectively [12], and it is also an inhibitor of human monoamine oxidase (hMAO) and a modulator of dopamine receptors [29]. Our results confirmed the strong inhibitory effects of kuwanon G on AChE and BChE with IC_50_ values of 19.8 and 5.7 μM. Combined with previous research, the present results indicate the promising potential of kuwanon G as a multi-target drug for neurodegenerative diseases. Kuwanon H (**13**), a further prenylated product of **12**, possessed even stronger inhibitory activity (BChE IC_50_ = 3.4 μM), indicating that the increase in prenyl groups in the molecule is conducive to enhancing molecular hydrophobicity, thereby improving its affinity for the binding site and elevating the enzyme inhibitory activity. Both compounds also exhibited favorable AChE inhibitory activities, with IC_50_ values of 19.8 μM and 17.5 μM, respectively.

Kuwanon X (**14**) is a Diels–Alder adduct of prenylated stilbenes and chalcone derivatives, and kuwanol A (**15**) is its intramolecular ketal cyclization product. Both compounds showed good BChE-inhibitory activity with IC_50_ values of 2.3 μM and 9.4 μM, respectively; however, **15** had reduced activity in comparison. This finding suggests that the further cyclization at the methylcyclohexene moiety may increase the rigidity of the molecular structure, which is unfavorable for enzyme binding. Mulberrofuran J (**16**) and mulberrofuran C (**17**) are Diels–Alder adducts of prenylated 2-phenylbenzofurans and chalcone derivatives, with all-trans and cis-trans configurations of the methylcyclohexene ring, respectively. Nevertheless, they exhibited almost equivalent inhibitory activities against both AChE and BChE. In contrast, mulberrofuran H (**18**), the product of debenzoylation and subsequent cyclization, showed no obvious inhibitory effect on either AChE or BChE, further confirming the essential role of the benzoyl group. Kuwanon X is the most potent BChE inhibitor discovered from Cortex Mori Radicis thus far, with slightly higher activity than our previously reported cathafuran C (IC_50_ = 2.6 μM) [11]. However, these BChE inhibitors from mulberry root bark remain moderately active relative to certain plant-derived natural products that inhibit BChE at nanomolar concentrations [4,5].

**Figure 5:**
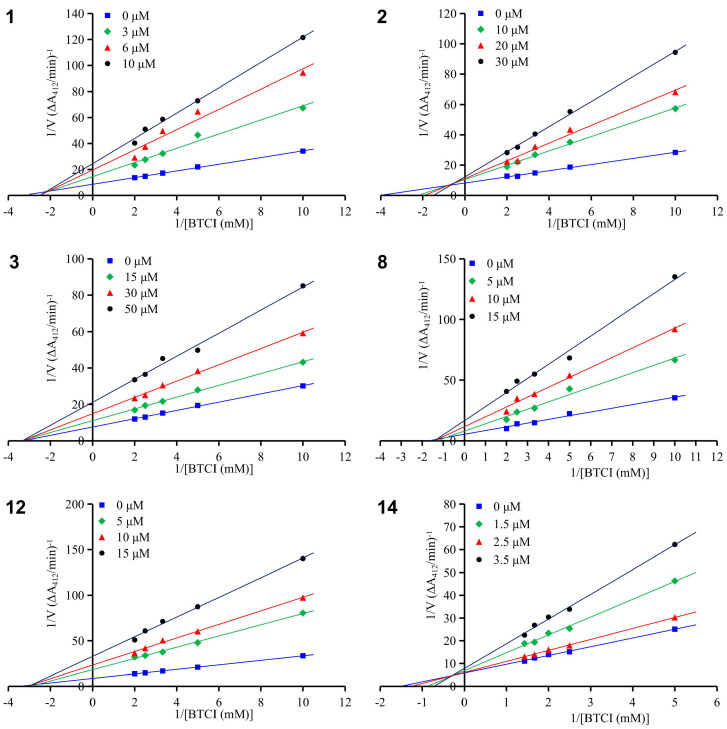
Lineweaver–Burk plots for BChE inhibition by compounds **1**–**3**, **8**, **12**, and **14**.

To determine the inhibition types of the compounds, enzyme kinetic studies were performed on the novel morusalbanol B–D (**1**–**3**) and the representative known compounds cathayanon A (**8**), sanggenon Q (**10**), kuwanon G (**12**), and kuwanon X (**14**). As shown in Figure 5, the Lineweaver–Burk double-reciprocal plots presented a set of straight lines with different slopes, all intersecting in the second quadrant or on the X-axis, indicating that all these compounds are mixed-type inhibitors. For this type of interaction, the inhibitor can bind to both the free enzyme (E) and the enzyme–substrate (ES) complex, forming EI and ESI complexes, respectively [30]. The Ki and αKi values of the inhibitors were determined by fitting the slope (K_m_/V_m_) and vertical intercept (1/V_m_) of the Lineweaver–Burk plots as functions of inhibitor concentration to quadratic curves, and the thermodynamic cooperativity factor α was subsequently calculated [31] (Table 2). Compound **2** had an α value of 5.82, indicating a higher affinity for the free enzyme E than for the ES complex, which is defined as mixed competitive inhibition (mixed competitive, α > 1). In contrast, **1** and **3** had α values of 1.39 and 0.99, respectively, and the double-reciprocal curves at different inhibitor concentrations intersected almost on the X-axis, demonstrating that both compounds exhibit nearly equal affinity for E and the ES complex, and thus exert their inhibitory effects via a noncompetitive mode (noncompetitive, α = 1). Similarly, the dihydroflavone or flavone-type Diels–Alder adducts **8** and **12** had α values close to 1, also acting as noncompetitive inhibitors. In addition, the stilbene-type Diels–Alder adduct **14** had an α value of 6.49, suggesting that this inhibitor preferentially binds to the free enzyme E and can be identified as a mixed competitive inhibitor of BChE.

### 2.3. Molecular Docking for Inhibitors with BChE

**Figure 6:**
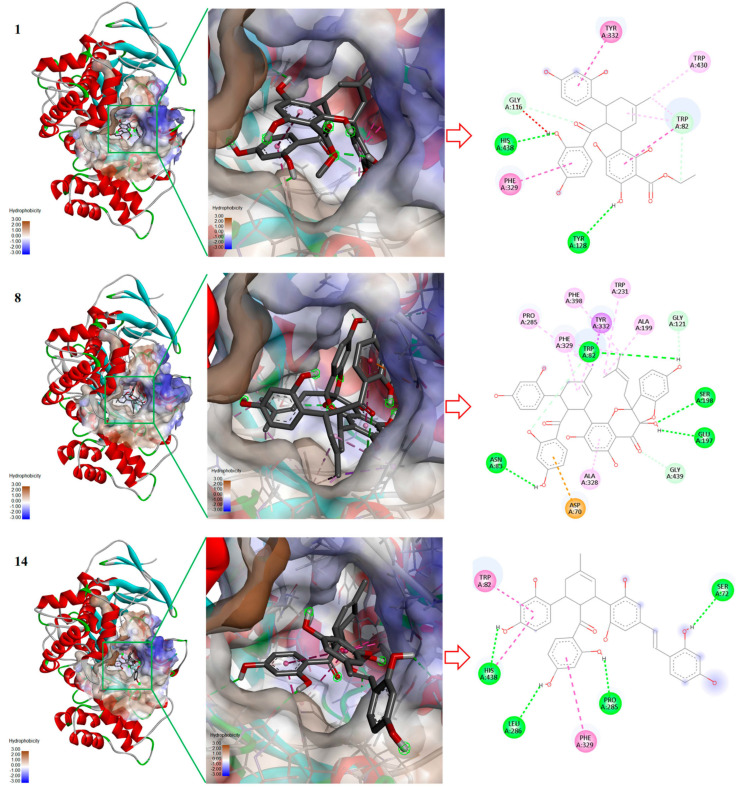
Molecular docking analysis of inhibitors **1**, **8**, and **14** in complex with BChE (PDB: 5k5e). Left: Overall binding pose of each inhibitor within the BChE active site, with the protein surface colored by hydrophobicity (brown: hydrophobic, blue: hydrophilic). Middle: 3D binding conformation of each inhibitor in the BChE catalytic pocket. Right: 2D interaction diagrams showing hydrogen bonds (green dashed lines), π-π stacking (pink dashed lines), and other noncovalent interactions between the inhibitors and key residues.

Analysis of the BChE inhibition mode revealed that more than 50% of the inhibitory effect of the above-mentioned compounds was competitive, which involves competing with the substrate for binding at the catalytic active site of the enzyme. The binding modes of **1**, **8**, and **14** with the enzyme active site were investigated using Autodock Vina v1.1.2 software. The active site of BChE is a gorge with a depth of approximately 20 Å, comprising the peripheral anionic site (PAS) at the edge of the active pocket, the acyl-binding pocket (Trp231, Leu286, and Val288), the catalytic triad (Ser198, His438, and Glu325), and the choline-binding pocket (Trp82) [32]. According to previous reports, the π-π interactions between inhibitor molecules and the amino acid residues Trp82, Trp231, and Phe329, as well as the hydrogen-bond interactions with His438, play a crucial role in BChE inhibition [32,33,34]. The results showed that all three compounds could be successfully inserted into the BChE binding pocket and form various interactions with enzyme residues (Figure 6). Compound **1** formed π-π stacking and π–alkyl interactions with the amino residue Trp82, π-π interactions with Phe329 and Tyr332, conventional hydrogen-bond interactions with Tyr128 and His438, and π–alkyl hydrophobic interactions with Trp430, indicating that it can occupy both the catalytic triad and the choline-binding pocket. Compound **8** formed hydrogen-bond interactions with Trp82, Asn83, Glu197, and Ser198, and hydrophobic interactions (π-π, π-σ, or π–alkyl) with multiple amino acid residues, including Trp82, Ala199, Trp231, Pro285, Ala328, Phe329, Tyr332, and Phe398; it also formed a π–anionic interaction with Asp70. These results indicated that the large spatial structure of **8** enables extensive and diverse interactions with amino acid residues in the catalytic pocket. However, its inhibitory activity was not substantially improved, presumably because the steric hindrance caused by the large molecular structure reduces the probability of its insertion into the active site, offsetting the binding advantages brought by the above interactions. Compound **14** has a molecular size between those of **1** and **8** and exhibited the strongest activity among the Diels–Alder adducts. Molecular docking showed that **14** exhibited the most extensive and balanced interaction network, combining strong π-π stacking with both TRP82 and PHE329, multiple hydrogen bonds with SER72, LEU286, and HIS438, and additional hydrophobic contacts. This multi-point binding mode anchored the inhibitor in the active site while occupying a large portion of the catalytic gorge.

### 2.4. Molecular Dynamics Simulation Stability Analyses

The molecular dynamics simulation results of the three inhibitor–BChE complexes are summarized in Figure S35. All systems underwent efficient energy minimization (Figure S35A), as evidenced by a rapid drop in potential energy followed by convergence to a stable low-energy state, confirming successful relaxation of initial steric clashes. During the production simulation (Figure S35B), the total energy, potential energy, kinetic energy, van der Waals energy, and electrostatic energy remained stable throughout the simulation for all three complexes, with no significant drifts or abrupt fluctuations, indicating that each system reached thermodynamic equilibrium under the simulation conditions. Structural stability was further evaluated by RMSD analysis (Figure S35C). For the complex with **1**, the BChE backbone RMSD remained low and stable (1.0–1.5 Å) throughout the 100 ns simulation, and the inhibitor heavy-atom RMSD showed only one major conformational rearrangement within the first 40 ns before stabilizing, suggesting a rigid and well-anchored binding mode. In contrast, the complex with **8** exhibited slower equilibration: the ligand RMSD fluctuated markedly during the first 40 ns, and the solute heavy-atom RMSD remained consistently higher than the backbone RMSD, indicating greater overall flexibility and a longer adaptation period before achieving a stable binding pose. For compound **14**, although the simulation duration was shorter (20 ns), the system still reached apparent equilibrium. The protein backbone stabilized at 1.0–1.5 Å, while the inhibitor heavy-atom RMSD showed the highest degree of fluctuation among the three compounds, reflecting a more dynamic and flexible binding interaction. Overall, all three inhibitors formed stable complexes with BChE. However, compound **1** demonstrated the highest structural stability, followed by **8**, while inhibitor **14** exhibited the greatest ligand flexibility within its shorter simulation time. These results support that all three compounds can bind stably to BChE, with inhibitor **1** showing the most favorable binding rigidity, which may contribute to its potential inhibitory activity.

**Figure 7:**
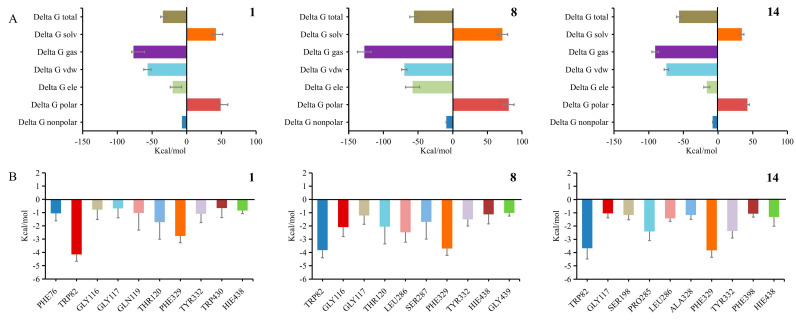
Total binding free energy decomposition and per-residue contribution analyses of inhibitors **1**, **8**, and **14** in complex with BChE (PDB: 5k5e) according to molecular dynamic simulation results: (**A**) Total binding free energy and its components, including van der Waals energy, electrostatic energy, polar solvation energy, nonpolar solvation energy, gas-phase energy, solvation energy, and total binding free energy. (**B**) Per-residue energy contributions for each inhibitor–BChE complex. Negative values indicate favorable binding contributions.

To quantitatively evaluate the binding affinity and interaction patterns of inhibitors **1**, **8**, and **14** with BChE (PDB: 5k5e), the total binding free energy and per-residue energy contributions were calculated using the MM-GBSA approach. As illustrated in Figure 7A, all three complexes yielded negative total binding free energies (ΔG total), verifying that these inhibitors can spontaneously bind to BChE under physiological conditions. Among them, compound **1** exhibited the most negative ΔG total, indicating the strongest binding affinity. Inhibitor **8** showed the most favorable gas-phase energy (ΔG gas), dominated by remarkable van der Waals contributions, yet its high polar desolvation penalty largely offset this advantage, resulting in a moderate overall binding free energy. Compound **14** displayed an energetic profile similar to **1** but with weaker electrostatic interactions, leading to a slightly less negative ΔG total. In all cases, van der Waals forces and nonpolar solvation energy represented the main favorable driving forces for binding, whereas polar solvation energy acted as the primary unfavorable component. Per-residue energy decomposition further revealed distinct binding modes (Figure 7B). Compound **1** relied on the strongest interaction with TRP82, followed by PHE329 and TYR332, while **8** presented dual dominant contributions from TRP82 and PHE329, indicating a broader hydrophobic interaction network. Compound **14** was mainly stabilized by PHE329 alongside strong interactions with TRP82, with additional favorable contributions from SER198, PRO285, and PHE398, representing a more dispersed binding pattern. TRP82, PHE329, and TYR332 were identified as conserved key hotspot residues across all three complexes.

The computational results were highly consistent with the in vitro IC_50_ data and molecular docking results. Although **1** had the most rigid and stable binding mode centered on TRP82, compound **14** exhibited the strongest activity, likely due to its combined use of both TRP82 and PHE329 as core anchoring points, paired with a more adaptive and extended interaction network that efficiently occupied the catalytic pocket despite moderate flexibility. Compound **8**, despite strong van der Waals interactions with both TRP82 and PHE329, showed the weakest activity among the three compounds that underwent molecular dynamics simulations, which could be attributed to its prolonged conformational adaptation caused by the large size of the molecule and high desolvation penalty. Collectively, the integrated analysis demonstrates that BChE inhibitory potency depends not only on structural rigidity and absolute binding free energy but also on adaptive binding dynamics and efficient pocket occupation through multiple hotspots. The high correlation between computational simulations and experimental data validates the reliability of our theoretical calculations and provides a robust molecular basis for understanding the mechanism of MDAAs as BChE inhibitors. These findings support the rational design of more potent BChE inhibitors that simultaneously engage key residues, such as TRP82 and PHE329, while optimizing energetic trade-offs and binding adaptability.

## 3. Materials and Methods

### 3.1. General Experimental Procedure

Column chromatography (CC) was carried out using AB-8 macroporous resin (Tianjin Yunkai Resin Technology Co., Ltd., Tianjin, China), silica gel (200–300 mesh, Qingdao Marine Chemical Inc., Qingdao, China), polyamide (60–100 mesh, Taizhou Luqiao Sijia Biochemical Plastic Factory, Taizhou, China), and YMC*GEL^®^ ODS-A-HG (12 nm, S-50 μm, YMC Co., Ltd., Kyoto, Japan) as the stationary phases. The fractions obtained from CC were analyzed by an Agilent 1100 HPLC system integrated with a photo-diode array detector (G1316A), which was equipped with an analytical Kromasil C-18 column (5 μm, 100 Å, 4.6 mm × 250 mm; Akzo Nobel, Amsterdam, The Netherlands). Preparative HPLC was conducted on a QuikSep chromatographic system (H&E, Beijing, China), with a Gemini C-18 column (21.2 mm × 250 mm) employed for the separation and purification process, and the column temperature was maintained at 26 °C. Optical rotations were determined using a P-2000 digital polarimeter (JASCO, Tokyo, Japan). UV spectra were measured with a UV-2600 spectrophotometer (Shimadzu, Kyoto, Japan). High-resolution electrospray ionization mass spectrometry (HR-ESI-MS) data were acquired on an Xevo G2-XS QTOF mass spectrometer (Agilent, Santa Clara, CA, USA), while nuclear magnetic resonance (NMR) spectra (500 MHz for ^1^H-NMR and 125 MHz for ^13^C-NMR) were recorded on a Bruker-500 spectrometer (Bruker, Berlin, Germany). A SynergyHTX microplate reader (BioTek, Winooski, VT, USA) was utilized to detect the absorbance values in the enzymatic activity assays. Acetylcholinesterase (AChE, EC 3.1.1.7, derived from electric eel), butyrylcholinesterase (BChE, EC 3.1.1.8, isolated from equine serum), acetylthiocholine iodide (ATCI), butyrylthiocholine iodide (BTCI), and 5,5′-dithiobis(2-nitrobenzoic acid) (DTNB) were all purchased from Aladdin Industrial Co., Ltd. (Shanghai, China).

### 3.2. Plant Material

Cortex Mori Radicis was purchased from the Bozhou Herb Market (Bozhou, China). The raw material was collected in Anhui Province, China, in 2020, and authenticated by Professor Jing Hu from the College of Traditional Chinese Medicine, Tianjin University of Traditional Chinese Medicine (Tianjin, China). A voucher specimen (voucher No. TM-2003) was deposited in the College of Life Sciences and Agronomy, Zhoukou Normal University.

### 3.3. Extraction and Isolation

The dried root bark of Cortex Mori Radicis (9.3 kg) was subjected to two rounds of reflux extraction using 80% aqueous ethanol (30 L) at 80 °C. Following filtration, the combined extracting solution was concentrated under reduced pressure at 60 °C to yield a 10% ethanol aqueous suspension (approximately 20 L) containing about 990 g of solid residues. Subsequently, the obtained suspension was loaded onto an AB-8 macroporous adsorption resin column with a column volume (CV) of 4 L, and gradient elution was performed sequentially with 10%, 30%, 50%, 60%, 80%, and 95% ethanol solutions, with each eluent volume set at 3 CVs. The eluate from 10% ethanol was collected as Fraction A (Fr-A, 580 g), the 30% ethanol eluate was designated as Fraction B (Fr-B, 61.2 g), the eluents eluted by 50–80% ethanol were combined to obtain Fraction C (Fr-C, 180 g), and the 95% ethanol eluate was defined as Fraction D (Fr-D, 50.8 g). Thereafter, Fr-C (180 g) was further separated by silica gel column chromatography (CC), and gradient elution was carried out with a dichloromethane–methanol (D-M) solvent system at volume ratios ranging from 100:0 to 70:30, affording four subfractions coded as Fr-C1 to Fr-C4. Fr-C2 (7.0 g, eluted by D-M 99:1–96:4) were subjected to ODS CC eluted by gradient aqueous methanol to obtain subfractions Fr-C21–Fr-C28. Fr-C25 (60% M eluate) was purified by preparative HPLC (69% M, 10 mL/min) to yield compound **4** (32 mg, *t*_R_ = 19 min). Fr-C3 (93 g, eluted by D-M 92:8–90:10) was subjected to polyamide CC by gradient elution with 40–90% E to obtain subtractions Fr-C31–Fr-C36. Fr-C33 (70% ethanol eluate) was separated by silica gel CC to obtain subfractions Fr-C331–Fr-C-334. Fr-C331 was separated by ODS CC, MPLC, and subsequently purified by preparative HPLC to obtain **18** (24 mg, *t*_R_ = 33 min, 60% M, 10 mL/min), **11** (56 mg, *t*_R_ = 39 min, 70% M, 10 mL/min). Fr-C332 was subjected to preparative HPLC with 70% M as the mobile phase (10 mL/min) to give compounds **3** (13 mg, *t*_R_ = 19.0 min), **1** (8 mg, *t*_R_ = 24.5 min), **6** (65 mg, *t*_R_ = 36.8 min), and **8** (52 mg, *t*_R_ = 46.0 min). Fr-C333 was further isolated by HPLC (gradient elution with 20%–70% M in 60 min) yielding **5** (34 mg, *t*_R_ = 15.2 min), **10** (18 mg, *t*_R_ = 22.5 min), **9** (69 mg, *t*_R_ = 25.1 min), **2** (13 mg, *t*_R_ = 26.4 min), **13** (16 mg, *t*_R_ = 31.7 min), and **7** (78 mg, *t*_R_ = 37.2 min). Fr-C334 contained one major component, which was subjected to preparative HPLC (67% M, 10 mL/min) to obtain **12** (1.2 g, *t*_R_ = 33.5 min). Fr-C35 (80% ethanol eluate) was separated by MPLC (gradient elution with 20–80% M in 40 min), affording **14** (9 mg, *t*_R_ = 15.7 min), **15** (7 mg, *t*_R_ = 21.8 min), **16** (23 mg, *t*_R_ = 24.5 min), and **17** (12 mg, *t*_R_ = 33.7 min), which were further purified by HPLC.

Morusalbanol B (**1**): pale yellow amorphous powder, UV (MeOH) λmax (logε): 220 (3.16), 275 (2.35), 320 (2.00) nm. [α]24D 158.3 (c 0.04, MeOH), CD (c 0.0004, MeOH) mdeg (nm): −4.22 (328), +14.64 (269), +16.60 (225), +26.92 (205). Positive HR-ESI-MS: m/z measured 537.1768 [M+H]^+^ (calculated for C_29_H_29_O_10_ 537.1761). For ^1^H and ^13^C NMR data, see Table 1.

Morusalbanol C (**2**): pale yellow amorphous powder, UV (MeOH) λmax (logε): 220 (3.21), 280 (2.20), 320 (2.03) nm. [α]24D 176.5 (c 0.04, MeOH), CD (c 0.00025, MeOH) mdeg (nm): −2.81 (326), +11.52 (269), +13.42 (223), +27.75 (205). Negative HR-ESI-MS: *m*/*z* measured 521.1448 [M−H]^−^ (calculated for C_28_H_25_O_10_ 521.1448). For ^1^H and ^13^C NMR data, see Table 1.

Morusalbanol D (**3**): pale yellow amorphous powder, UV (MeOH) λmax (logε): 220 (3.26), 275 (2.31), 320 (1.98) nm. [α]24D −136.1 (c 0.05, MeOH), CD (c 0.0004, MeOH) mdeg (nm): −2.72 (325), −11.74 (285), +4.26 (267), −26.36 (212). Negative HR-ESI-MS: *m*/*z* measured 535.1605 [M−H]^−^ (calculated for C_29_H_27_O_10_ 535.1604). For ^1^H and ^13^C NMR data, see Table 1.

Morusalbanol E (**4**): pale yellow amorphous powder, UV (MeOH) λmax (logε): 223 (3.14), 275 (2.19), 312 (1.22) nm. [α]24D 36.8 (c 0.03, MeOH), CD (c 0.0003, MeOH) mdeg (nm): +0.36 (312), +6.42 (282), −5.95 (247), +2.01 (227), −8.16 (211), +25.89 (200). Positive HR-ESI-MS: *m*/*z* measured 399.1437 [M+H]^+^ (calculated for C_22_H_23_O_7_ 399.1444). For ^1^H and ^13^C NMR data, see Table 1.

### 3.4. ECD Calculation

Systematic random conformational searches were carried out for compound **4** and its enantiomer ***ent*-4** using the MMFF94s force field within the SYBYL 8.1 software package. Conformers within an energy window of 7 kcal/mol relative to the global minimum were retained, and redundant conformers were removed using an RMSD threshold of 0.5 Å, yielding four stable conformers for each enantiomer. Each conformer was optimized with ORCA 4.2 using density functional theory (DFT) sequentially at the PM3, B97-3C, and B3LYP/def2-TZVP(-f) levels with a net charge of 0 and spin multiplicity of 1, employing the CPCM polarizable continuum solvent model throughout all optimization steps. Subsequent time-dependent density functional theory (TDDFT) calculations were performed at the wB97X/def2-SV(P) level to simulate the excited-state properties, with the first 30 electronic excitations included. Theoretical ECD spectra were constructed by Boltzmann-weighted averaging of the individual conformer contributions, applying a half-bandwidth of 0.4 eV. Simulated ECD curves for **4** and ***ent*-4** were then compared with the experimental ECD data. All calculated ECD spectra were processed and plotted using SpecDis 1.6 software.

### 3.5. ChE Inhibitory Activity Assay

The inhibitory effects of compounds **1**–**18** on acetylcholinesterase (AChE, EC 3.1.1.7) and butyrylcholinesterase (BChE, EC 3.1.1.8) were evaluated via the Ellman’s method [35], with minor modifications based on our previously published research [11]. In detail, solutions of AChE and BChE (each at 0.2 units/mL), acetylthiocholine iodide (ATCI), butyrylthiocholine iodide (BTCI), and 5,5′-dithiobis-(2-nitrobenzoic acid) (DTNB) (each at 10 mM) were prepared using phosphate-buffered saline (PBS, 0.1 M, pH 8.0). Stock solutions of the test compounds were prepared in methanol at a concentration of 10 mM, and five serial double dilutions of each compound were further prepared with methanol to determine the half-maximal inhibitory concentration (IC_50_) values. The assay procedure was conducted as follows: initially, 160 μL of PBS, 2 μL of the test sample, 20 μL of AChE or BChE solution, and 10 μL of DTNB solution were mixed thoroughly and preincubated at 37 °C for 10 min. Subsequently, 10 μL of ATCI or BTCI was added to trigger the enzymatic reaction, which was then incubated at 37 °C for 25 min. During the incubation period, the absorbance of the reaction mixture was measured at a wavelength of 412 nm. The inhibition rate (IR) was calculated using the following formula: IR% = [(Ac − As)/(Ac − Ab)] × 100%, where Ab refers to the absorbance of the blank control (20 μL of water instead of the enzyme solution), Ac represents the absorbance of the negative control (2 μL of methanol instead of the test sample), and As denotes the absorbance of the test sample. All experiments for each sample were performed in triplicate to ensure reproducibility. The IC_50_ value of each compound was calculated by plotting the inhibition rate against the logarithm of its concentration.

### 3.6. Kinetic Study of BChE Inhibition

Kinetic studies on BChE inhibition were carried out following the same experimental protocol as the inhibitory activity assay described above. Specifically, a series of substrate BTCI concentrations was employed, set as either 0.1, 0.2, 0.3, 0.4, and 0.5 mM or 0.2, 0.4, 0.5, 0.6, and 0.7 mM. For each test compound, three different concentrations were selected based on its corresponding IC_50_ value. During the enzymatic reaction, absorbance measurements were taken at three time points: 10 min, 20 min, and 25 min. The enzyme-catalyzed reaction velocity was defined as the change in absorbance per minute. Lineweaver–Burk plots (double-reciprocal plots) were constructed for each compound at its three selected concentrations by plotting the reciprocal of the reaction velocity (1/V) against the reciprocal of the BTCI concentration (1/[BTCI]).

### 3.7. Molecular Docking and Molecular Dynamics Simulations

Docking calculations were employed to explore the binding modes of the BChE–inhibitor complexes, with all simulations performed using AutoDock Vina v1.1.2 software [36]. The detailed experimental procedure was consistent with our previously published work [11]. Briefly, the three-dimensional (3D) structures of the test compounds were constructed first, followed by energy minimization using the MM2 force field via Chem3D Ultra 2017 (Version 17.0.0.206) until a minimum Root Mean Square (RMS) gradient of 0.005 was achieved. The crystal structures of BChE (PDB codes: 5k5e) were retrieved from the Protein Data Bank (PDB) and subjected to further preparation, which involved the removal of water molecules, ions, and native ligands. Subsequently, using AutoGrid, the grid box was set with a dimension of 26 × 26 × 26 Å, and its center was positioned at the coordinates (x = 2.967, y = 4.171, z = 9.571), corresponding to the Trp82 residue. All parameters for simulated annealing were kept at their default settings. After the completion of docking simulations, nine top-ranked ligand–receptor conformations were obtained, which were sorted based on the calculated binding free energy. The optimal conformation of each ligand, characterized by the highest affinity score (expressed in kcal/mol), was visualized using Discovery Studio Visualizer v25.1.0.24284 (Accelrys, San Diego, CA, USA) to analyze the interaction patterns between the enzyme and respective inhibitors.

Molecular dynamics (MD) simulations of the inhibitor–enzyme complex were performed utilizing the PMEMD module within AMBER 20. For structural optimization, the protein was parameterized with the AMBER ff99SB force field, while the general AMBER force field was adopted for ligand pretreatment [37]. The assembled simulation systems were immersed in a 10 Å cubic TIP3P water box, and additional sodium ions were supplemented to maintain overall charge neutrality. Two successive energy minimization rounds (1000 steps for each run) were conducted to alleviate steric hindrance and structural tension. Subsequently, the systems were gradually warmed to 300 K in an NVT ensemble for 20 ps via the Langevin thermostat, with a mild restraint of 10 kcal/mol/Å^2^ imposed on protein backbone atoms. Next, 1 ns of NPT ensemble equilibration was carried out at 1 atm and 300 K with the Langevin thermostat, followed by 2 ns of NVT ensemble equilibrium using the Berendsen thermostat. Ultimately, trajectory snapshots collected from a 20 ns simulation were exported for subsequent analysis via CPPTRAJ [38]. Meanwhile, the MMGBSA approach embedded in AMBER 20 was employed to compute the total binding free energy and dissect individual energy contributions [39].

## 4. Conclusions

This study performed a systematic phytochemical and pharmacological investigation on Cortex Mori Radicis, isolating eighteen Diels–Alder adducts and elucidating the structures of four new ones (morusalbanol B–E, **1**–**4**) by multi-spectroscopic analysis. Pharmacological tests confirmed the selective BChE-inhibitory potential of most isolates, among which kuwanon X (**14**) displayed superior activity to the clinical drug galantamine. Enzyme kinetics clarified two main inhibition modes of active compounds. Molecular docking, molecular dynamics simulation, and binding free energy analysis jointly revealed that active compounds stably bound to the BChE active pocket, with Trp82 and Phe329 as key hotspot residues and binding driven primarily by van der Waals forces. Moreover, this study identified key structural factors that regulate BChE-inhibitory activity, providing clear structure–activity relationship clues for subsequent compound optimization. In summary, this work enriches the structural library of mulberry Diels–Alder adducts, screens out promising lead compounds for anti-AD BChE-inhibitor development, and provides in-depth dynamic molecular mechanisms for their inhibitory effects. These findings lay a solid foundation for the further development and utilization of Cortex Mori Radicis as a natural medicinal resource for neurodegenerative disease treatment.

## Figures and Tables

**Table 1:** ^1^H NMR (500 MHz, J in Hz) and ^13^C NMR (125 MHz) data of **1**–**4** (δ in ppm) ^a^.

No.	1		2		3		4	
	δ_C_	δ_H_	δ_C_	δ_H_	δ_C_	δ_H_	δ_C_	δ_H_
1	94.2 s	—	94.2 s	—	94.2/93.5 s	—	93.9 s	—
2	162.3 s	—	162.3 s	—	162.8/161.3 s	—	160.6 s	—
3	108.5 s	—	108.5 s	—	109.5 s	—	110.8 s	—
4	161.8 s	—	161.7 s	—	161.8/161.6 s	—	162.7 s	—
5	97.1 d	5.78 s	97.2 d	5.79 s	96.6/95.5 d	5.83/5.66 s	95.8 d	5.83 s
6	164.5 s	—	164.5 s	—	165.1/163.6 s	—	162.3 s	—
7	171.3 s	—	171.8 s	—	171.2/171.4 s	—	171.3 s	—
8	33.6 d	4.11 d (6.4)	33.6 d	4.10 br s	39.0/38.7 d	4.34/4.27 br d (8.2)	135.3 s	—
9	124.2 d	5.52 br s	124.2 d	5.53 br s	126.0/125.8 d	5.26 br s	132.1 d	5.40 br s
10	134.4 s	—	134.4 s	—	133.5 s	—	72.5 s	—
11	34.1 t	Hα 2.46 dd (17.7, 5.0) Hβ 2.20 br d (17.2)	34.0 t	Hα 2.45 dd (17.2, 5.5) Hβ 2.19 br d (17.2)	ND	Hα 2.52 br s Hβ 2.13 br d (14.7)	35.3 t	Hα 2.67 ddd (17.2, 4.4, 2.3) Hβ 2.27 br d (17.2)
12	36.0 d	3.77 q (5.0)	36.1 d	3.77 q (5.5)	ND	3.57 br s	32.5 d	3.07 brs
13	48.4 d	4.45 t (5.0)	48.4 d	4.44 t (5.5)	47.4 d	ND	39.9 t	1.96 dd (12.6, 2.2) 1.87 dd (12.6, 4.0)
14	23.8 d	1.86 br s	23.8 d	1.86 br s	23.5 d	1.72 br s	27.8 d	1.50 br s
15	123.4 s	—	123.3 s	—	122.8 s	—	120.0 s	—
16	156.9 s	—	156.9 s	—	157.0 s	—	155.8 s	—
17	103.6 d	6.30 d (2.4)	103.6 d	6.30 d (2.4)	103.8 d	6.12 br s	103.9 d	6.17 d (2.4)
18	157.6 s	—	157.7 s	—	157.2 s	—	157.5 s	—
19	107.3 d	6.19 dd (8.4, 2.4)	107.3 d	6.19 dd (8.4, 2.4)	107.3 d	5.99/6.05 br d (8.3)	108.6 d	6.29 dd (8.2, 2.4)
20	129.2 d	6.88 d (8.4)	129.2 d	6.88 d (8.4)	132.8 d	6.83/6.81 d (8.3)	130.7 d	6.88 d (8.2)
21	209.6 s	—	209.6 s	—	210.9 s	—	—	—
22	114.5 s	—	114.4 s	—	116.2 s	—	—	—
23	166.7 s	—	166.7 s	—	165.8 s	—	—	—
24	103.4 d	6.13 d (2.3)	103.4 d	6.14 d (2.4)	102.8 d	5.95 d (2.3)	—	—
25	166.3 s	—	166.4 s	—	165.6 s	—	—	—
26	108.7 d	6.26 dd (8.9, 2.3)	108.7 d	6.27 dd (8.9, 2.4)	108.1/107.9 d	6.05 br d (7.8)	—	—
27	135.0 d	8.16 (d, *J* = 8.9 Hz, 1H)	135.0 d	8.20 d (8.9)	134.2 d	7.62 br s	—	—
1′	63.0 t	4.44 q (7.0)	52.7 q	3.91 s	63.0 t	4.52/4.41 q (7.0)	63.1 t	4.45 q (7.1)
2′	14.6 q	1.37 t (7.0)	—	—	14.7/14.5 q	1.44/1.36 t (7.0)	14.6 q	1.37 t (7.1)

^a^ Tested in methanol-*d*_4_ and chemical shift values were recorded using the solvent signals at δ_C_ 49.00 and δ_H_ 3.31 as references. ND: not detected.

**Table 2:** The inhibitory activities against ChEs of compounds **1**–**18**.

Cpd.	IC_50_ ^a^ μM		BChE			
	AChE	BChE	Ki ^b^ μM	αKi μM	α	Inhibition Type
**1**	>100	6.5	4.2	5.8	1.39	noncompetitive
**2**	>100	19.6	11.3	65.7	5.82	mixed competitive
**3**	>100	28.3	26.3	26.2	0.99	noncompetitive
**4**	>100	>100	—	—	—	—
**5**	>100	13.8	—	—	—	—
**6**	>100	11.7	—	—	—	—
**7**	29.6	10.9	—	—	—	—
**8**	30.5	9.5	5.5	6.5	1.19	noncompetitive
**9**	38.6	12.3	—	—	—	—
**10**	>100	19.3	—	—	—	—
**11**	>100	27.9	—	—	—	—
**12**	19.8	5.7	5.3	5.9	1.11	noncompetitive
**13**	17.5	3.4	—	—	—	—
**14**	15.6	2.3	1.5	9.8	6.49	mixed competitive
**15**	55.3	9.4	—	—	—	—
**16**	41.2	23.6	—	—	—	—
**17**	48.2	24.8	—	—	—	—
**18**	>100	>100	—	—	—	—
Galantamine ^c^	0.8	35.3	—	—	—	—

^a^ Sample concentration that led to 50% enzyme activity loss. ^b^ Ki is the inhibition constant. ^c^ Galantamine is used as a positive control. — not tested.

## Data Availability

The data presented in this study are available in the Supplementary Materials.

## Supplementary Materials

The following supporting information can be downloaded at: https://www.mdpi.com/article/10.3390/molecules31101574/s1, Figures S1–S33: HRESIMS, ^1^H NMR, ^13^C NMR, ^1^H-^1^H COSY, HSQC, HMBC, ROESY, UV, and ECD spectra of compounds **1**–**4**; Figure S34: Low-energy conformation of compound **4** optimized by DFT; Figure S35: Molecular dynamics simulation stability analyses of the inhibitor–BChE complexes.

## Abbreviations

The following abbreviations are used in this manuscript:

AD	Alzheimer’s disease
ACh	Acetylcholine
AChE	Acetylcholinesterase
ATCI	Acetylthiocholine iodide
BChE	Butyrylcholinesterase
BTCI	Butyrylthiocholine iodide
ChE	Cholinesterase
CC	Column chromatography
DEPT	Distortionless Enhancement by Polarization Transfer
DFT	Density functional theory
DTNB	5,5′-dithiobis-(2-nitrobenzoic acid)
ECD	Electronic circular dichroism
^1^H-^1^H COSY	^1^H-^1^H Correlation Spectroscopy
HMBC	Heteronuclear Multiple Bond Correlation
HSQC	Heteronuclear Single Quantum Coherence
HR-ESI-MS	High-resolution electrospray ionization mass spectrometry
IC_50_	Half-maximal inhibitory concentration
IHD	Index of hydrogen deficiency
MDAAs	Mulberry Diels–Alder-type adducts
NMR	Nuclear magnetic resonance
NPs	Natural products
PAS	Peripheral anionic site
PBS	Phosphate-buffered saline
SAR	Structure–activity relationship
TCM	Traditional Chinese Medicine
UV	Ultraviolet

## References

[1] Park S., Son S.J., Park K., Nam Y., Shin H. (2024). In-house data adaptation to public data: Multisite MRI harmonization to predict Alzheimer’s disease conversion. Expert Syst. Appl..

[2] Goedert M., Spillantini M.G. (2006). A century of Alzheimer’s disease. Science.

[3] Li Q., Xing S., Chen Y., Liao Q., Xiong B., He S., Lu W., Liu Y., Yang H., Li Q. (2020). Discovery and biological evaluation of a novel highly potent selective butyrylcholinsterase inhibitor. J. Med. Chem..

[4] Khan U., Ahmad M., Tuba M., Naaz R., Qayum F., Khatoon S., Sanobar, Ahamad S., Saquib M., Hussain M.K. (2026). Natural alkaloids with therapeutic potential against Alzheimer’s disease through cholinesterase inhibition. Eur. J. Med. Chem..

[5] Hostalkova A., Marikova J., Opletal L., Korabecny J., Hulcova D., Kunes J., Novakova L., Perez D.I., Jun D., Kucera T. (2019). Isoquinoline alkaloids from Berberis vulgaris as potential lead compounds for the treatment of Alzheimer’s disease. J. Nat. Prod..

[6] Zhang B., Yu H., Li W., Yu B., Liu L., Jia W., Lin Z., Wang H., Chen S. (2019). Four new honokiol derivatives from the stem bark of *Magnolia officinalis* and their anticholinesterase activities. Phytochem. Lett..

[7] Özbek H., Güvenalp Z., Yılmaz G., Yerdelen K.Ö., Kazaz C., Demirezer Ö.L. (2018). In vitro anticholinesterase activity and molecular docking studies of coumarin derivatives isolated from roots of *Heptaptera cilicica*. Med. Chem. Res..

[8] Chan E.W.C., Lye P.Y., Wong S.K. (2016). Phytochemistry, pharmacology, and clinical trials of *Morus alba*. Chin. J. Nat. Med..

[9] Luo S.Y., Zhu J.Y., Zou M.F., Yin S., Tang G.H. (2022). Mulberry Diels-Alder-type adducts: Isolation, structure, bioactivity, and synthesis. Nat. Prod. Bioprospect..

[10] Tortora C., Pisano L., Vergine V., Ghirga F., Iazzetti A., Calcaterra A., Marković V., Botta B., Quaglio D. (2022). Synthesis, biosynthesis, and biological Activity of Diels-Alder adducts from Morus Genus: An update. Molecules.

[11] Cui X., Huang Z., Deng S., Zhang Y., Li G., Wang L., Deng Y., Wu C. (2024). Benzofuran derivatives from Cortex Mori Radicis and their cholinesterase-inhibitory activity. Molecules.

[12] Kuk E.B., Jo A.R., Oh S.I., Sohn H.S., Seong S.H., Roy A., Choi J.S., Jung H.A. (2017). Anti-Alzheimer’s disease activity of compounds from the root bark of *Morus alba* L.. Arch. Pharm. Res..

[13] Shen R.C., Lin M. (2001). Diels-Alder type adducts from *Morus cathayana*. Phytochemistry.

[14] Lu X., Su Y., Qiu L., Wu P. (2002). Separation of sanggenon D from Sangbaipi. Nat. Prod. Res. Dev..

[15] Taro N., Jin Y.S., Yoshio H. (1989). On the structure of sanggenon Q, a new Diels-Alder type adduct from *Morus mongolica* Schneider. Heterocycles.

[16] Hano Y., Itoh M., Fukai T., Urano S. (1985). Revised structure of sanggenon B. Heterocycles.

[17] Taro N., Toshio F. (1980). Kuwanon G, a new flavone derivative from the root barks of the cultivated Mulberry tree (*Morus alba* L.). Chem. Pharm. Bull..

[18] Cui X., Wang H., Liu C., Chen R. (2008). Study of anti-oxidant phenolic compounds from stem barks of *Morus yunanensis*. China J. Chin. Mater. Medica.

[19] Hirakura K., Hano Y., Fukai T., Nomura T., Uzawa J., Fukushima K. (1985). Structures of three new natural Diels-Alder type adducts, kuwanons P and X, and mulberrofuran J, from the cultivated mulberry tree (*Morus lhou* Koidz.). Chem. Pharm. Bull..

[20] Nomura T., Hano Y., Itoh M. (1985). Structures of kuwanols A and B, two novel stilbene derivatives from the cultivated Mulberry tree (*Morus bombycis* Koidz.). Heterocycles.

[21] Wu D., Zhang X., Huang X., He X., Wang G., Ye W. (2010). Chemical constituents from *Morus atropurpurea*. China J. Chin. Mater. Medica.

[22] Nomura T., Fukai T., Hano Y., Hirakura K., Uzawa J., Fukushima K. (1984). Structure of mulberrofuran H, a novel 2-arylbenzofuran derivative from the cultivated mulberry tree (*Morus lhou* (ser.) koidz.). Chem. Pharm. Bull..

[23] Nomura T. (1988). Phenolic compounds of the Mulberry tree and related plants. Fortschritte Chem. Org. Naturstoffe.

[24] Zhao X., Qiu Z., Ma Z., Liu Y., Ren X., Yu X., Sun L., Wang M. (2022). Comprehensive quality evaluation of the root bark of *Morus alba* L. Based on high-performance liquid chromatography fingerprinting and chemometric analyses. Chem. Biodivers..

[25] Yang L., Zhao F., Zhang T., Guo F., Lu H. (2016). Isolation of active compounds from Cortex Mori and its mechanism on anti-inflammatory. Chin. Arch. Tradit. Chin. Med..

[26] Jung H.W., Kang S.Y., Kang J.S., Kim A.R., Woo E.R., Park Y.K. (2014). Effect of kuwanon G isolated from the root bark of *Morus alba* on ovalbumin-induced allergic response in a mouse model of asthma. Phytother. Res..

[27] Jin S.E., Ha H., Shin H.K., Seo C.S. (2019). Anti-allergic and anti-inflammatory effects of kuwanon G and morusin on MC/9 mast cells and HaCaT keratinocytes. Molecules.

[28] Liu X.X., Zhang X.W., Wang K., Wang X.Y., Ma W.L., Cao W., Mo D., Sun Y., Li X.Q. (2018). Kuwanon G attenuates atherosclerosis by upregulation of LXRα-ABCA1/ABCG1 and inhibition of NF-κB activity in macrophages. Toxicol. Appl. Pharmacol..

[29] Paudel P., Park S.E., Seong S.H., Jung H.A., Choi J.S. (2019). Novel Diels-Alder type adducts from *Morus alba* root bark targeting human monoamine oxidase and dopaminergic receptors for the management of neurodegenerative diseases. Int. J. Mol. Sci..

[30] Copeland R.A. (2000). Enzymes: A Practical Introduction to Structure, Mechanism, and Data Analysis.

[31] Buker S.M., Boriack-Sjodin P.A., Copeland R.A. (2019). Enzyme-inhibitor interactions and a simple, rapid method for determining inhibition modality. SLAS Discov..

[32] Nicolet Y., Lockridge O., Masson P., Fontecilla-Camps J.C., Nachon F. (2003). Crystal structure of human butyrylcholinesterase and of its complexes with substrate and products. J. Biol. Chem..

[33] Nachon F., Carletti E., Ronco C., Trovaslet M., Nicolet Y., Jean L., Renard P.Y. (2013). Crystal structures of human cholinesterases in complex with huprine W and tacrine: Elements of specificity for anti-Alzheimer’s drugs targeting acetyl- and butyrylcholinesterase. Biochem. J..

[34] Knez D., Brus B., Coquelle N., Sosič I., Šink R., Brazzolotto X., Mravljak J., Colletier J.P., Gobec S. (2015). Structure-based development of nitroxoline derivatives as potential multifunctional anti-Alzheimer agents. Bioorg. Med. Chem..

[35] Ellman G.L., Courtney K.D., Andres V.J., Featherstone R.M. (1961). A new and rapid colorimetric determination of acetylcholines-terase activity. Biochem. Pharmacol..

[36] Trott O., Olson A.J. (2010). AutoDock Vina: Improving the speed and accuracy of docking with a new scoring function, efficient optimization, and multithreading. J. Comput. Chem..

[37] Hornak V., Abel R., Okur A., Strockbine B., Roitberg A., Simmerling C. (2006). Comparison of multiple Amber force fields and development of improved protein backbone parameters. Proteins.

[38] Roe D.R., Cheatham T.E. (2013). PTRAJ and CPPTRAJ: Software for processing and analysis of molecular dynamics trajectory data. J. Chem. Theory Comput..

[39] Ylilauri M., Pentikäinen O.T. (2013). MMGBSA as a tool to understand the binding affinities of filamin-peptide interactions. J. Chem. Inf. Model..

