# Supplementary material for: Phytochemical Profiling of Mulberry Diels-Alder Adducts as Selective Butyrylcholinesterase Inhibitors: In Vitro Activity, Molecular Docking, and Molecular Dynamics Simulation
Source: Molecules. 2026 May 8;31(10):1574. doi: 10.3390/molecules31101574 (PMC13209706; doi:10.3390/molecules31101574)
Supplement: Supplementary file 1 [file molecules-31-01574-s001.zip › molecules-4273826-supplementary.pdf]

Supplementary Materials

Phytochemical Profiling of Mulberry Diels-Alder Adducts as Selective Butyrylcholinesterase Inhibitors: In Vitro Activity, Molecular Docking, and Molecular Dynamics Simulation

Xiang Cui[1,2,3], Xiu-Cheng Zhu[1], Shu-Qi Yao[1], Rui Wang[1], Yun-Xia Zhang[1,2], Jin Li[1,2], Biao Wang[1,2], Yan-Ru Deng[3]* and Chang-Jing Wu[1,2,4]*

[1] College of Life Sciences and Agronomy, Zhoukou Normal University, Zhoukou 466001, China
[2] Fuxi Laboratory, Zhoukou Normal University, Zhoukou 466001, China
[3] College of Traditional Chinese Medicine, Tianjin University of Traditional Chinese Medicine, Tianjin 301617, China
[4] Field Observation and Research Station of Green Agriculture in Dancheng County, Zhoukou 466001, China
* Correspondence: dengyanru@tjutcm.edu.cn (Y.-R.D.); wucj2009@163.com (C.-J.W.)

List of Supplementary Materials

Figure S1. HR-ESI-MS spectrum of compound 1 1
Figure S2. ^{1}H NMR spectrum of compound 1 in CD_3OD 1
Figure S3. ^{13}C-NMR and DEPT spectra of compound 1 in CD_3OD 2
Figure S4. HSQC spectrum of compound 1 2
Figure S5. ^{1}H-^{1}H COSY spectrum of compound 1 3
Figure S6. HMBC spectrum of compound 1 3
Figure S7. ROESY spectrum of compound 1 4
Figure S8. UV spectrum of compound 1 4
Figure S9. ECD spectrum of compound 1 5
Figure S10. HR-ESI-MS spectrum of compound 2 6
Figure S11. ^{1}H-NMR spectrum of compound 2 in CD_3OD 6
Figure S12. ^{13}C-NMR spectrum of compound 2 in CD_3OD 7
Figure S13. HSQC spectrum of compound 2 7
Figure S14. ^{1}H-^{1}H COSY spectrum of compound 2 8
Figure S15. HMBC spectrum of compound 2 8
Figure S16. UV spectrum of compound 2 9
Figure S17. ECD spectrum of compound 2........ 9
Figure S18. HR-ESI-MS spectrum of compound 3 10
Figure S19. ^{1}H-NMR spectrum of compound 3 in CD_3OD 10
Figure S20. ^{13}C-NMR and DEPT spectra of compound 3 in CD_3OD 11
Figure S21. HSQC spectrum of compound 3 11
Figure S22. ^{1}H-^{1}H COSY spectrum of compound 3 12
Figure S23. HMBC spectrum of compound 3 12
Figure S24. UV spectrum of compound 3 13
Figure S25. ECD spectrum of compound 3 13
Figure S26. HR-ESI-MS spectrum of compound 4 14
Figure S27. ^{1}H-NMR spectrum of compound 4 in CD_3OD 14
Figure S28. ^{13}C-NMR spectrum of compound 4 in CD_3OD 15
Figure S29. HSQC spectrum of compound 4 15
Figure S30. ^{1}H-^{1}H COSY spectrum of compound 4 16
Figure S31. HMBC spectrum of compound 4 16
Figure S32. UV spectrum of compound 4 17
Figure S33. ECD spectrum of compound 4 17
Figure S34. Low-energy conformation of compound 4 optimized by DFT 18
Figure S35. Molecular dynamics simulation stability analyses of the inhibitors-BChE complex. 18

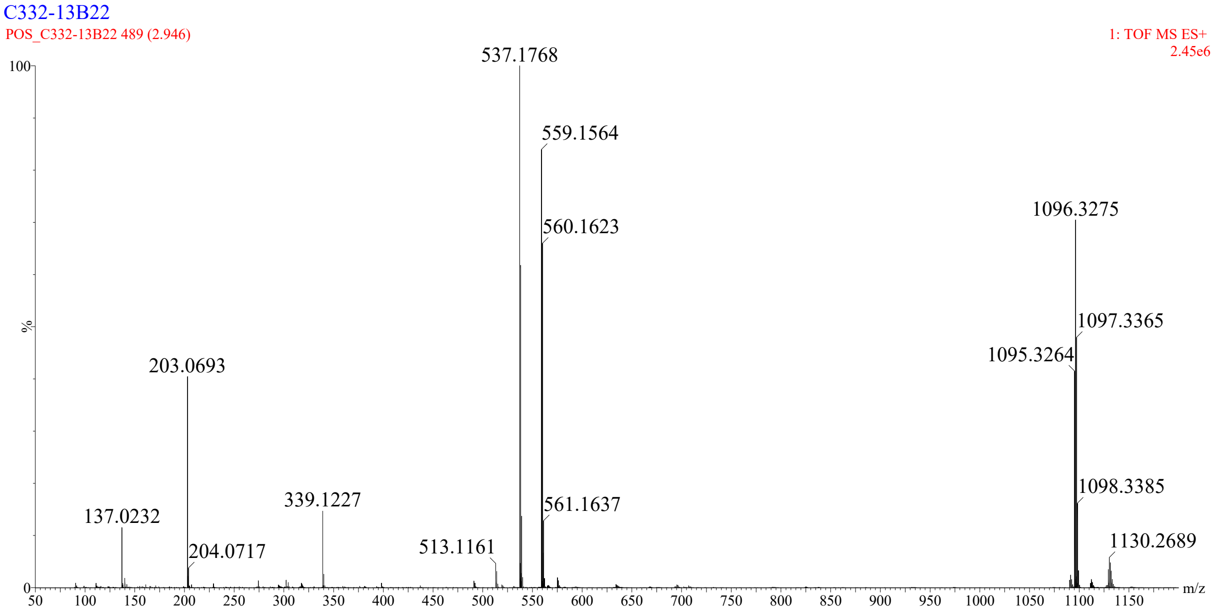

Figure S1. HR-ESI-MS spectrum of compound **1**

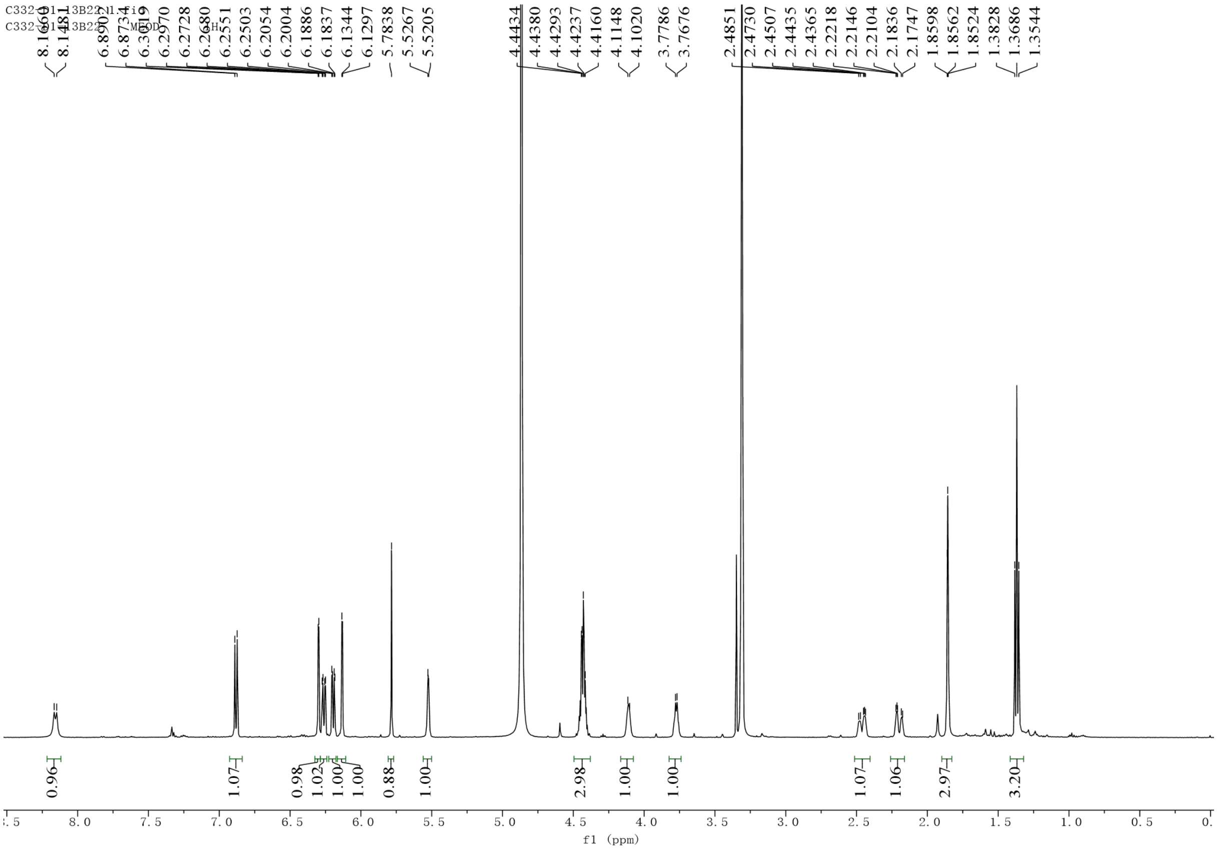

Figure S2. ^{1}H NMR spectrum of compound **1** in CD_3OD

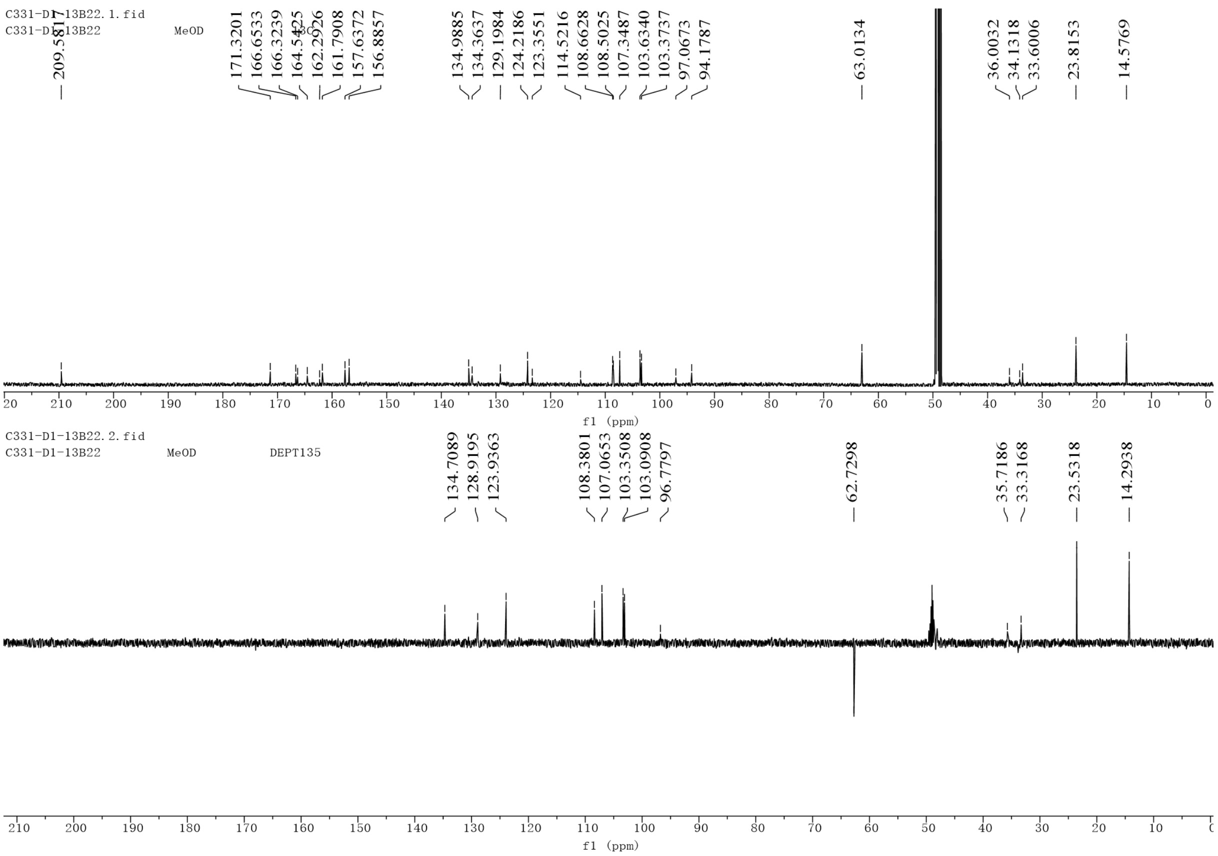

Figure S3. ^{13}C-NMR and DEPT spectra of compound **1** in CD_3OD

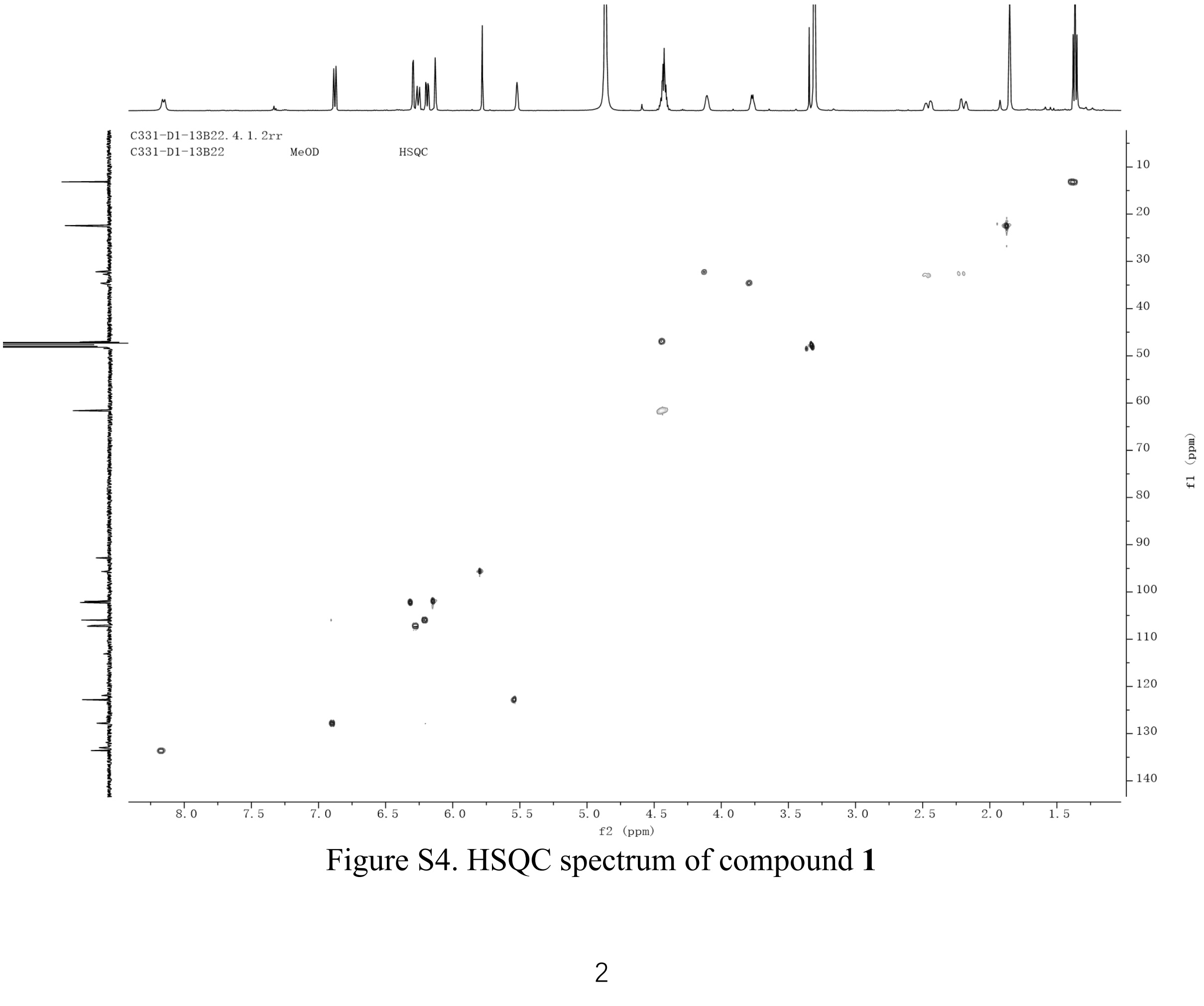

Figure S4. HSQC spectrum of compound **1**

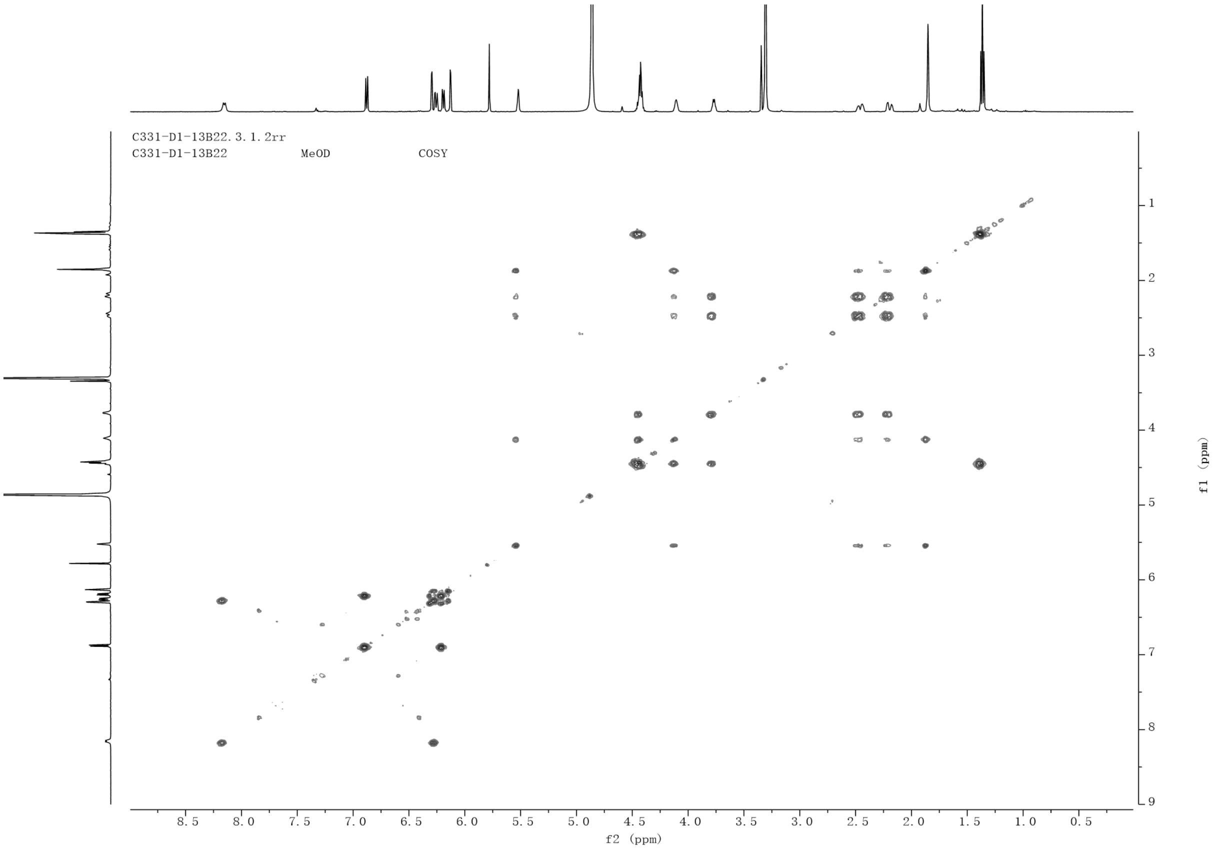

Figure S5. ^{1}H-^{1}H COSY spectrum of compound **1**

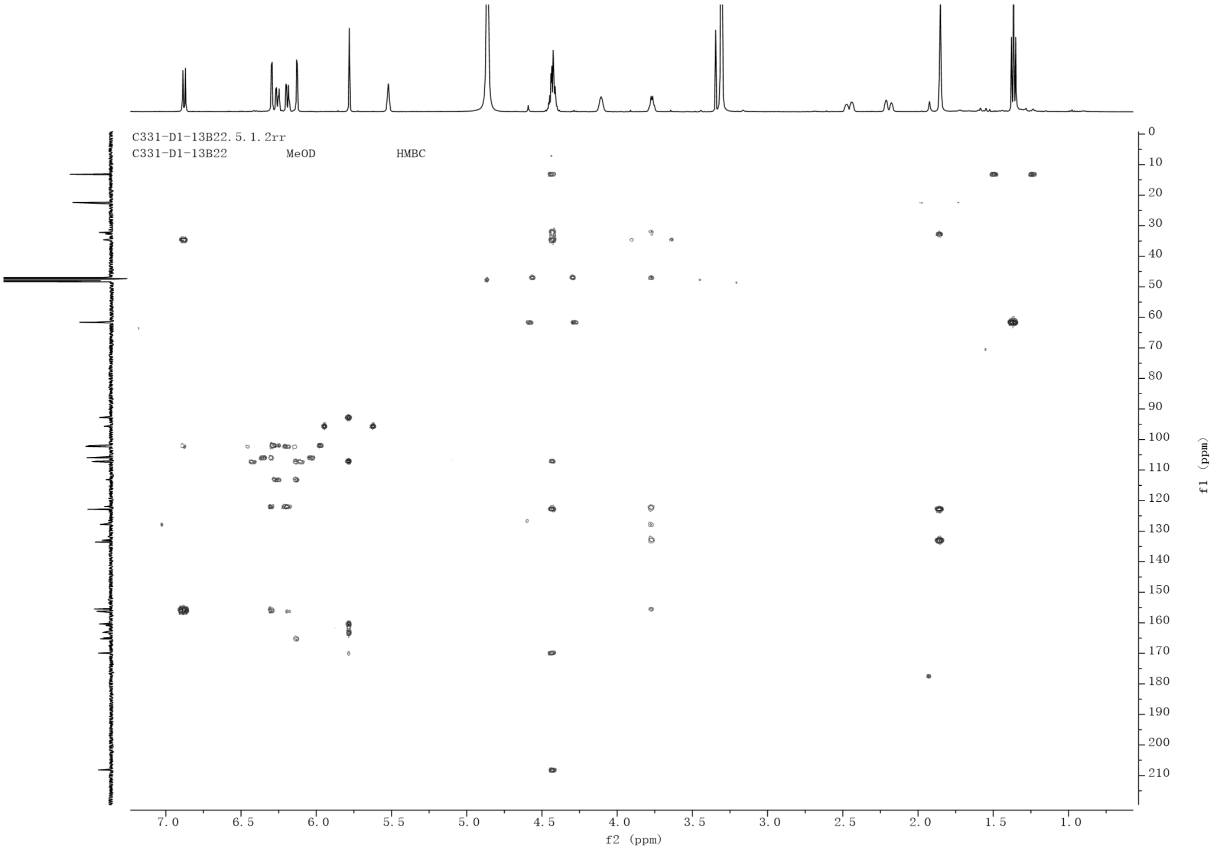

Figure S6. HMBC spectrum of compound **1**

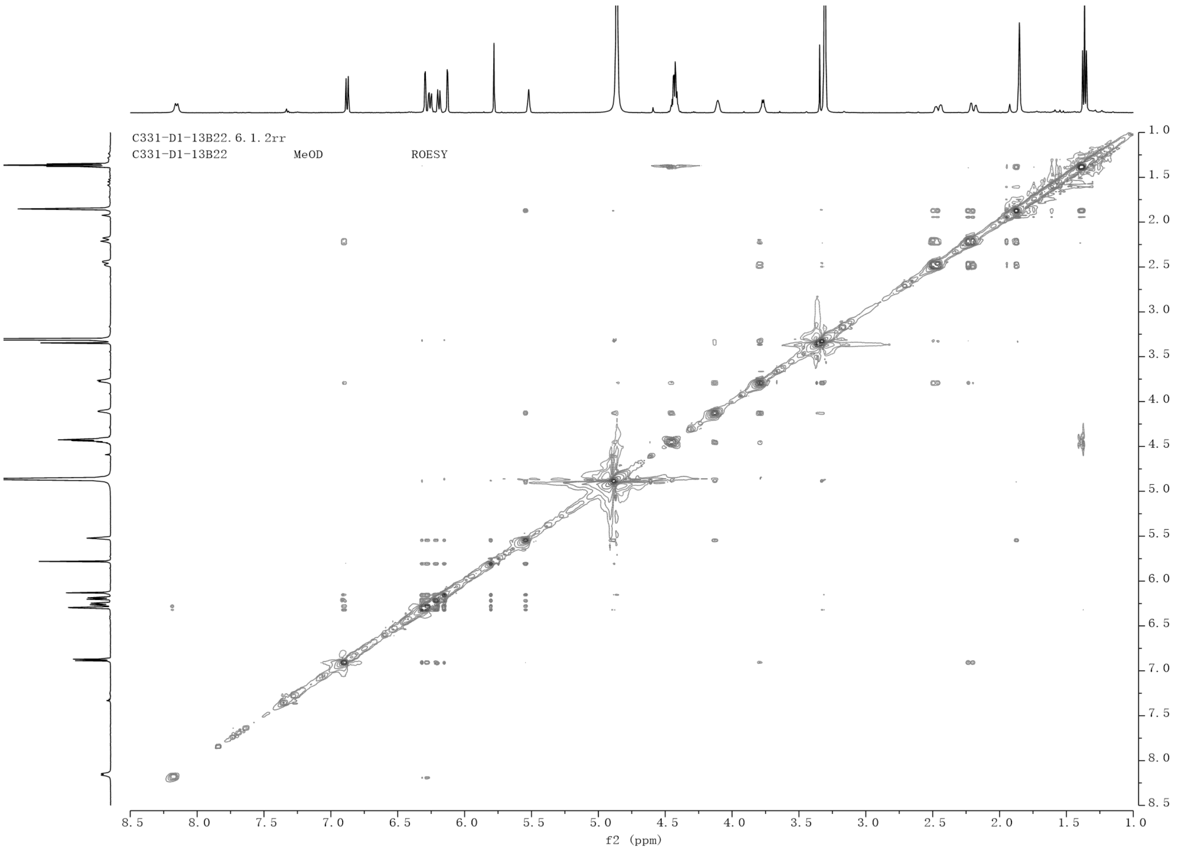

Figure S7. ROESY spectrum of compound **1**

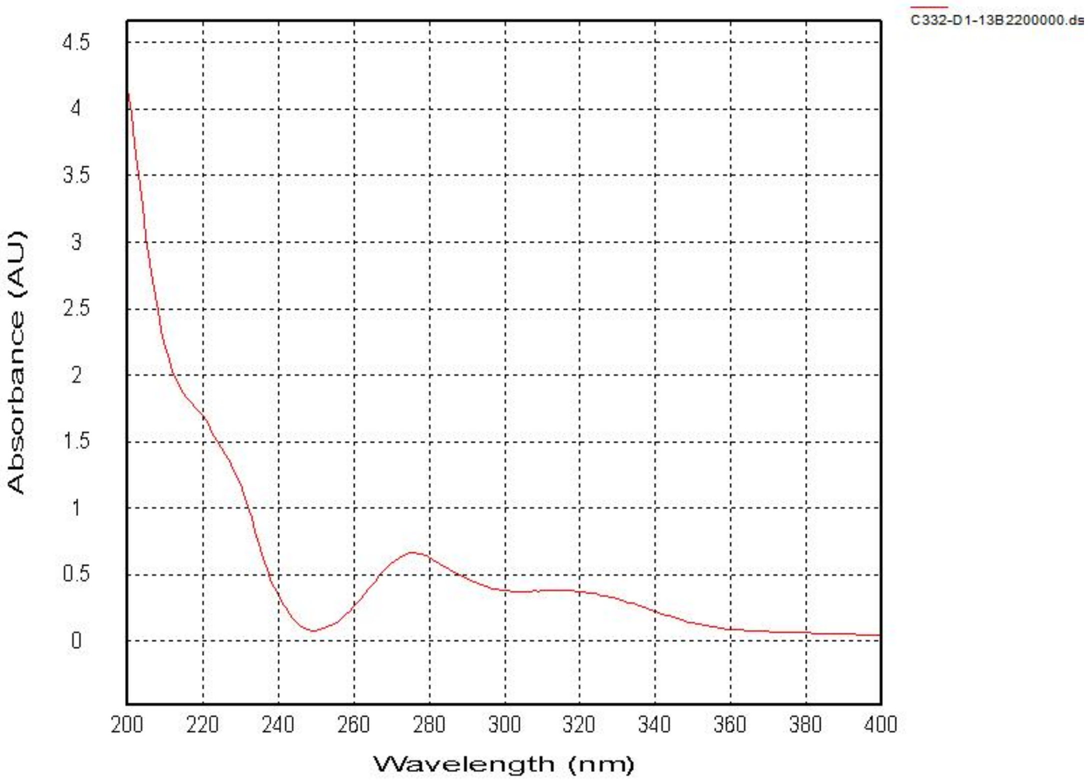

Figure S8. UV spectrum of compound **1**

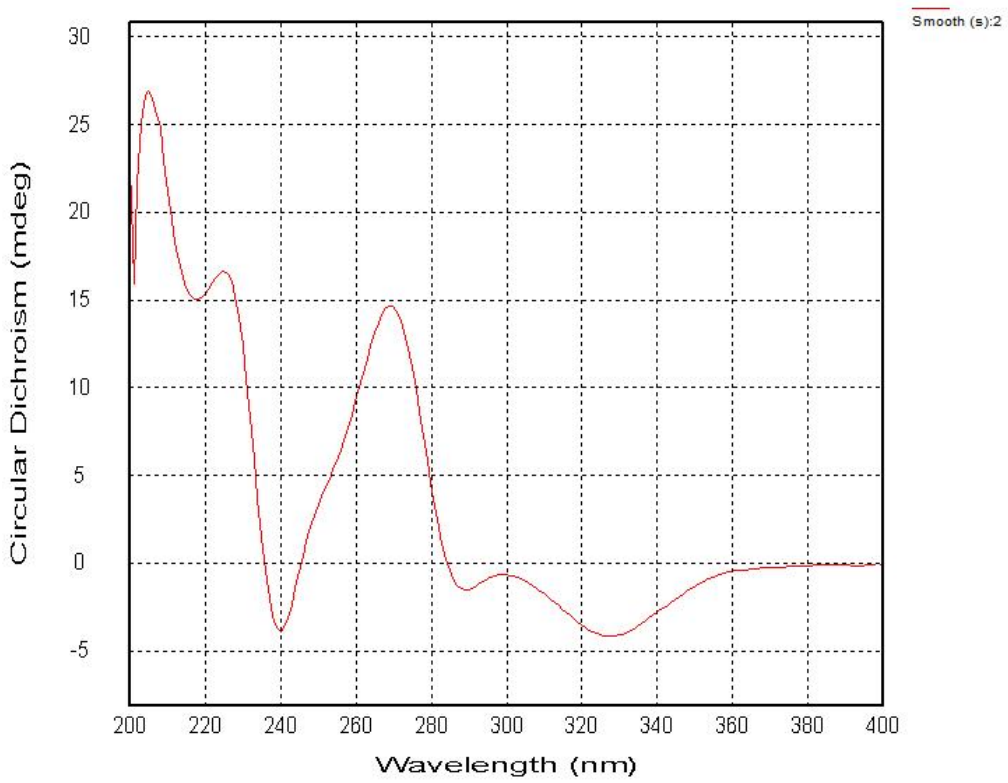

Figure S9. ECD spectrum of compound **1**

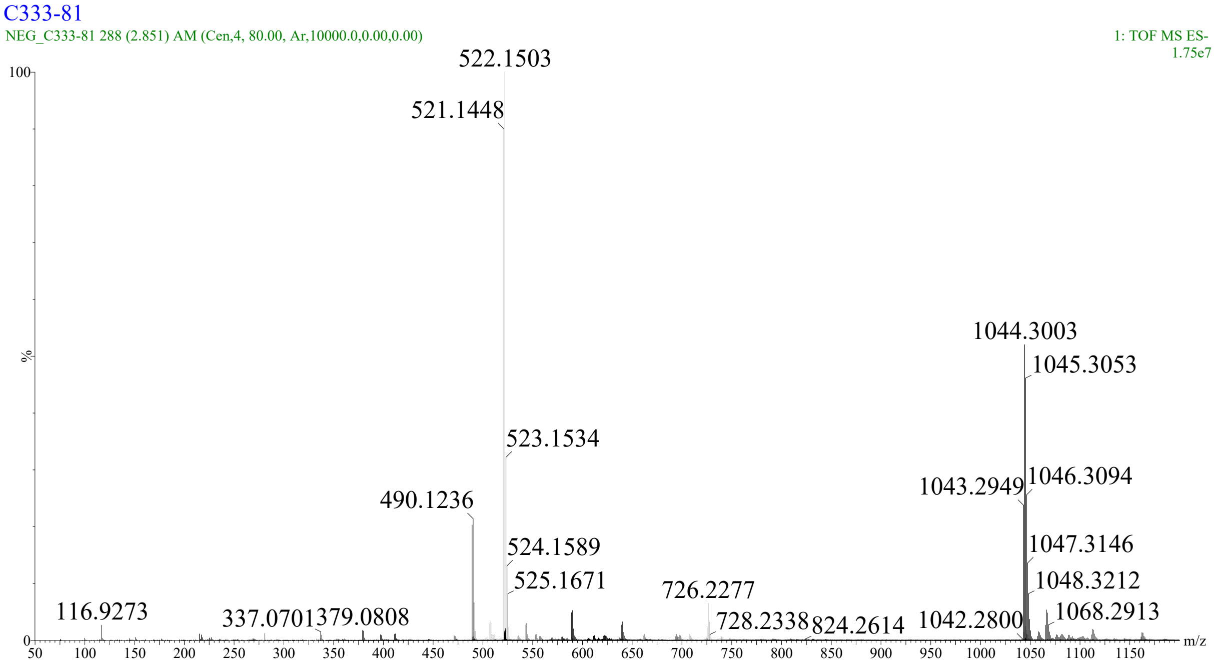

Figure S10. HR-ESI-MS spectrum of compound **2**

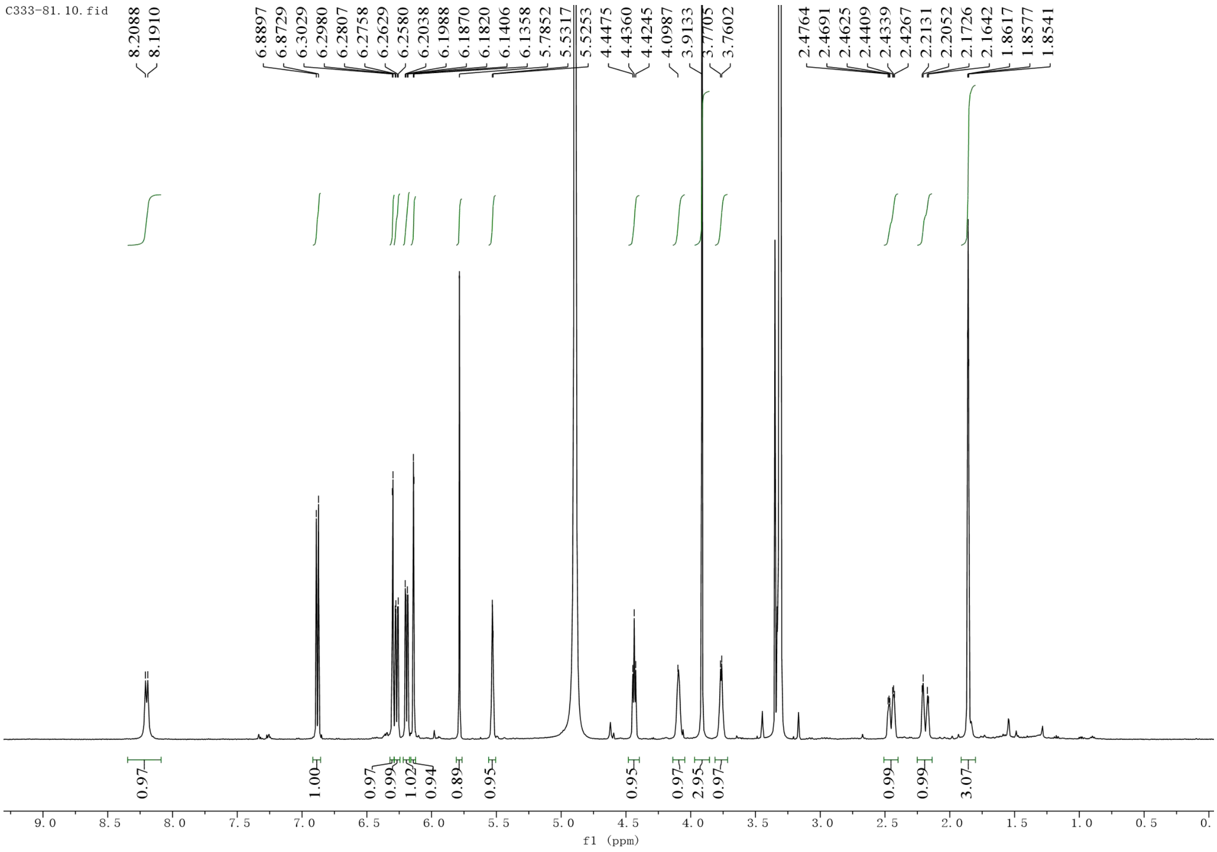

Figure S11. ^{1}H-NMR spectrum of compound **2** in CD_3OD

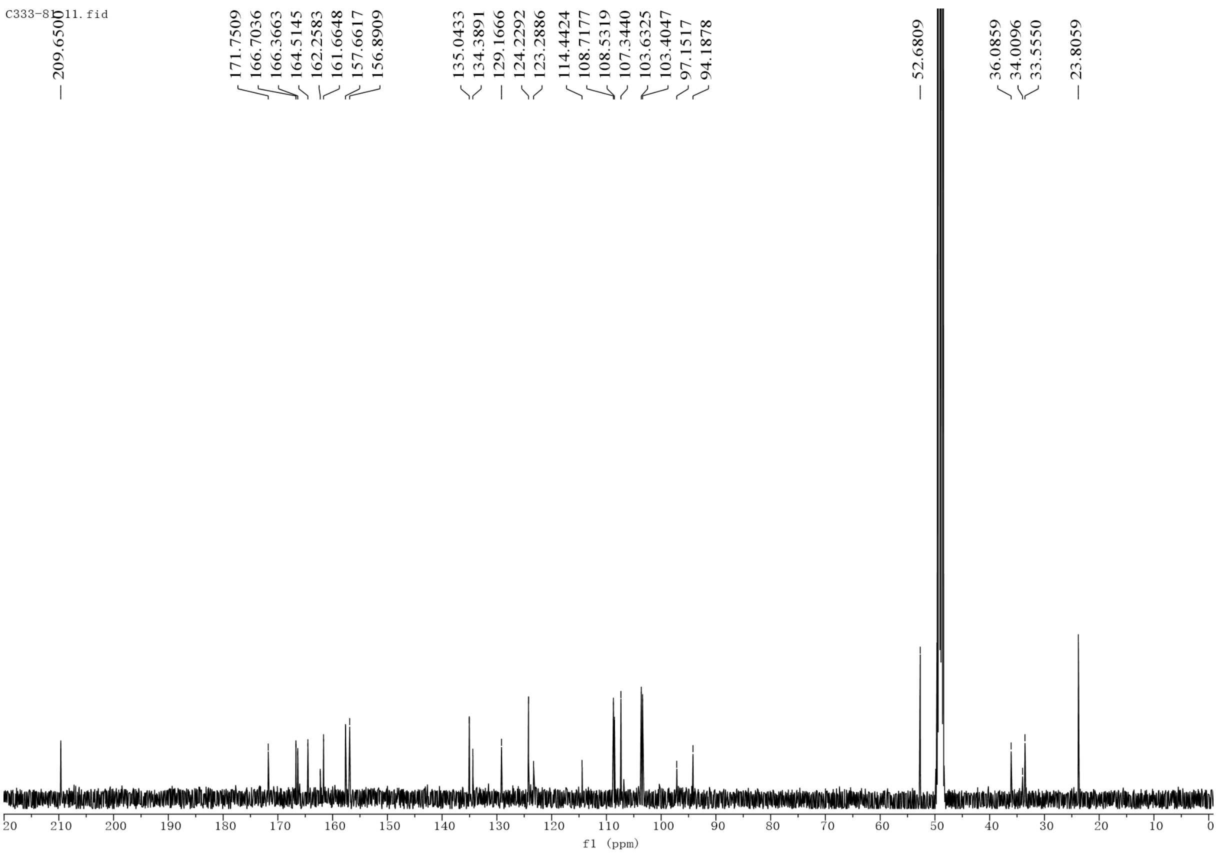

Figure S12. ^{13}C-NMR spectrum of compound **2** in CD_3OD

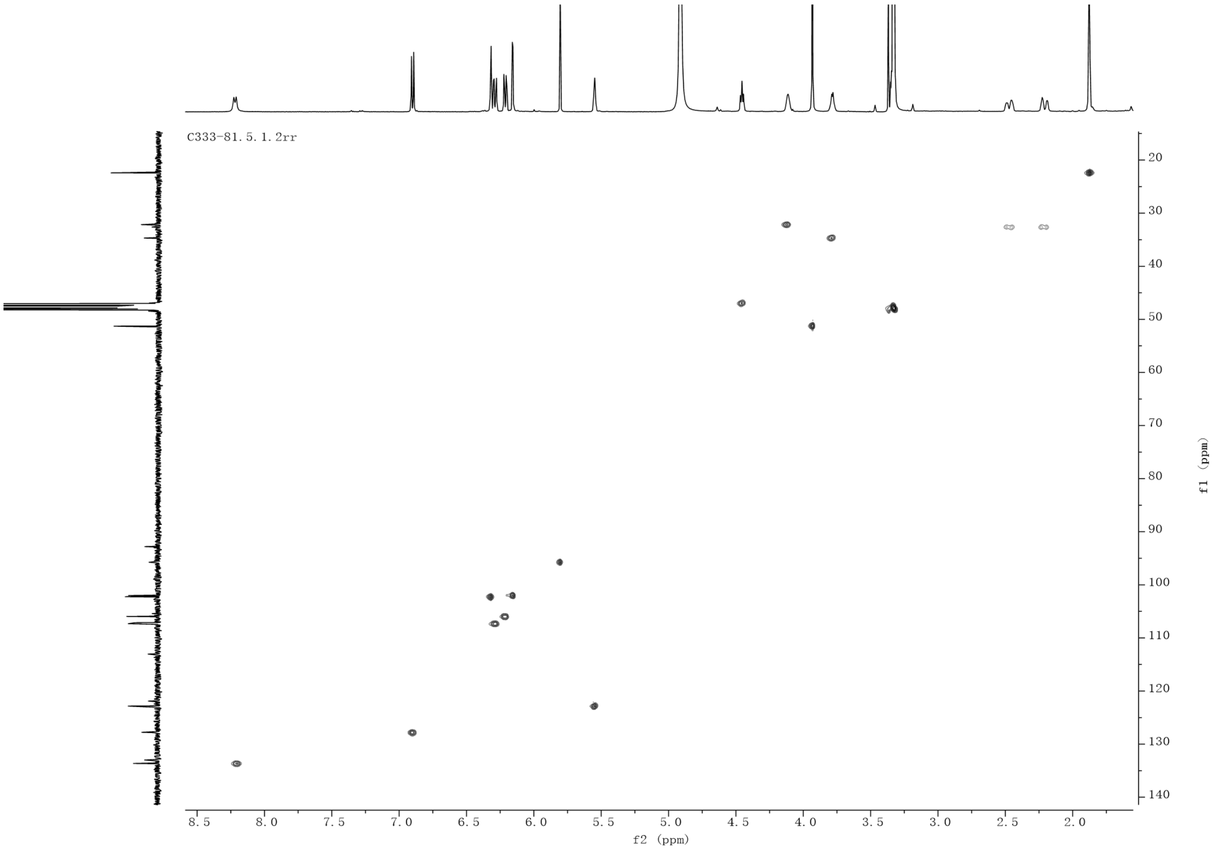

Figure S13. HSQC spectrum of compound **2**

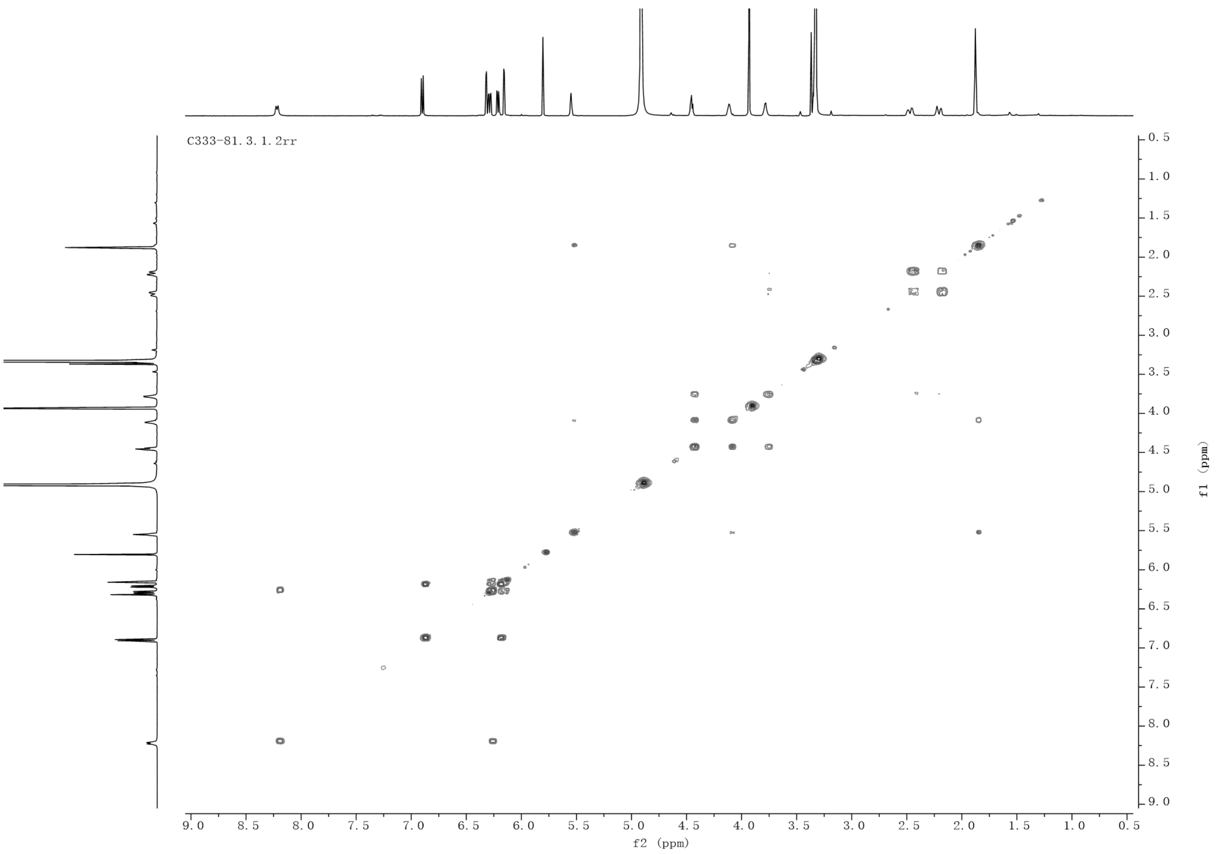

Figure S14. ^{1}H-^{1}H COSY spectrum of compound **2**

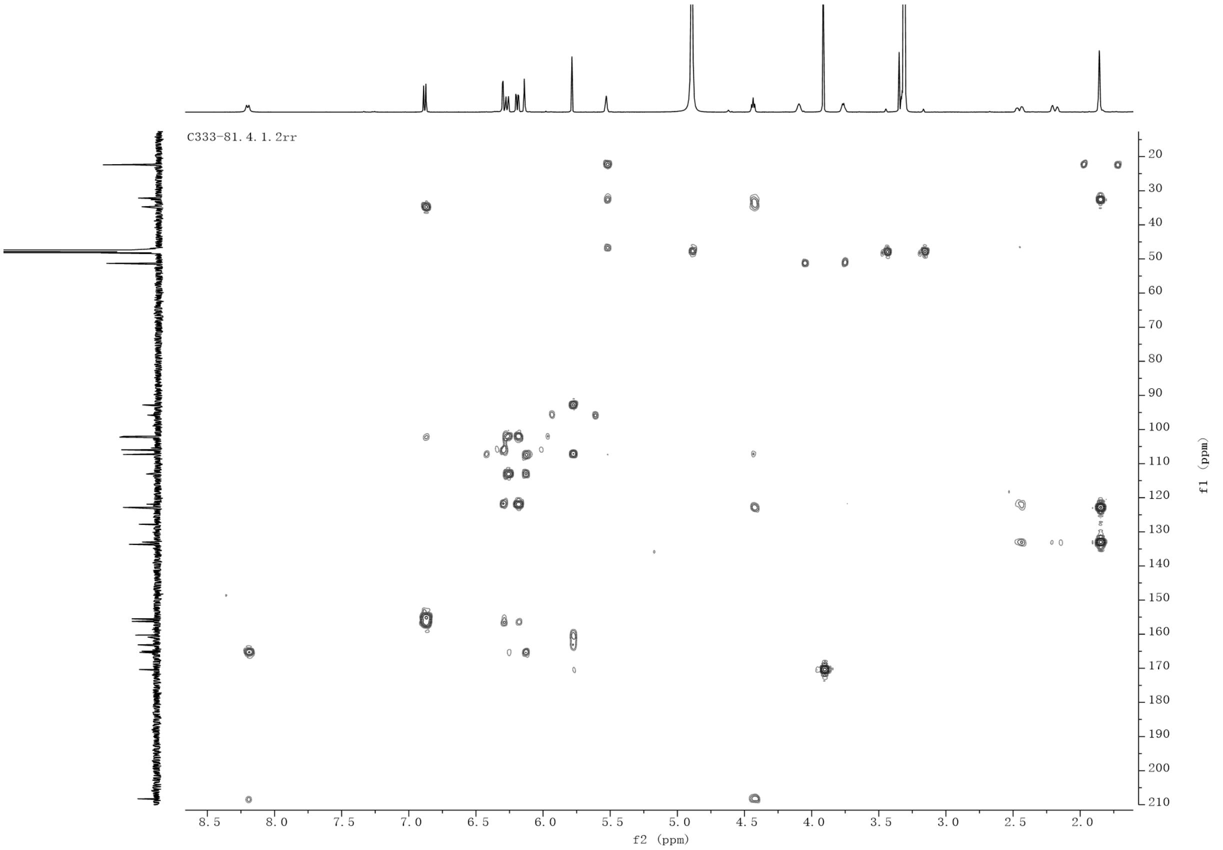

Figure S15. HMBC spectrum of compound **2**

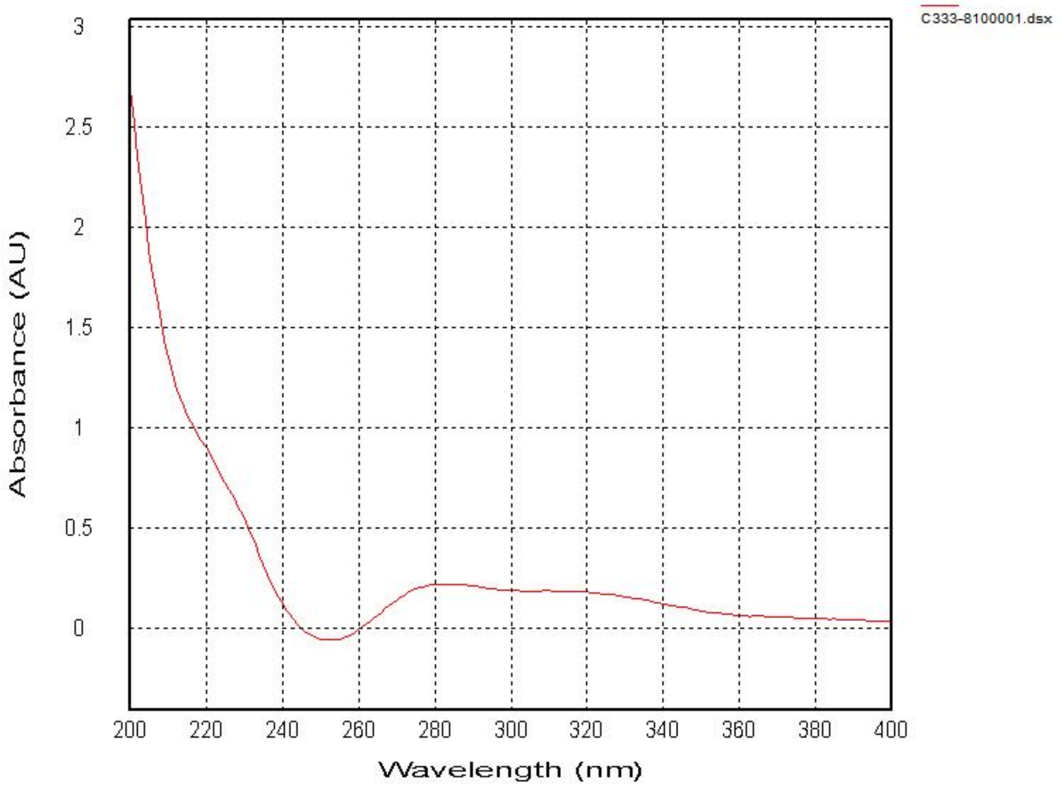

Figure S16. UV spectrum of compound **2**

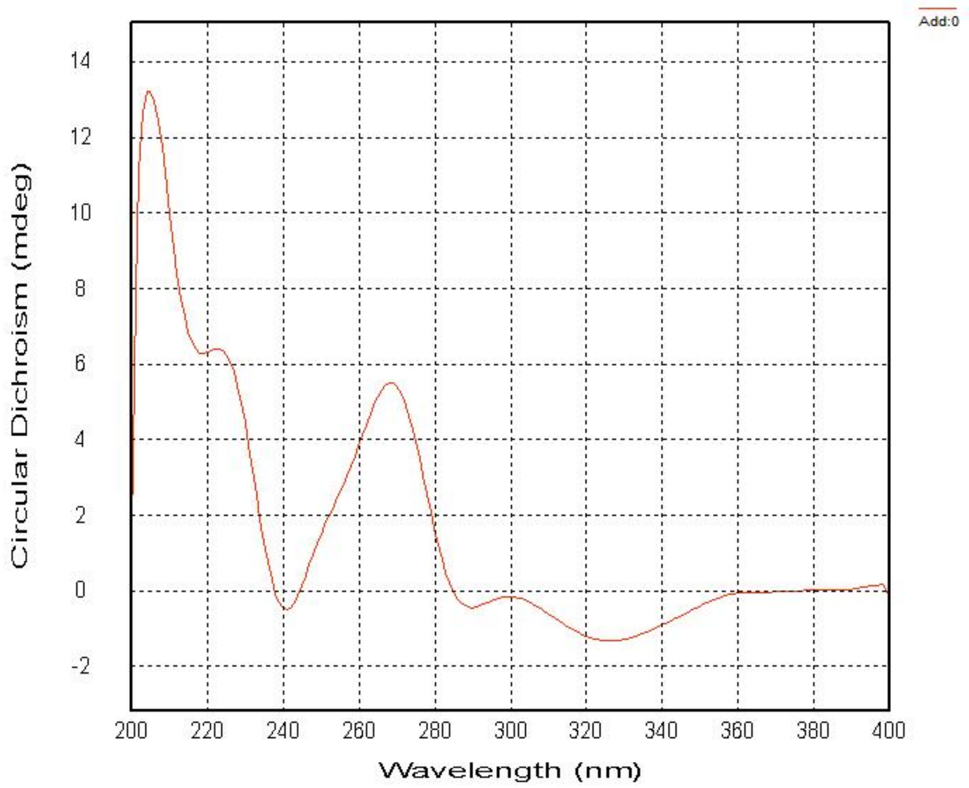

Figure S17. ECD spectrum of compound **2**

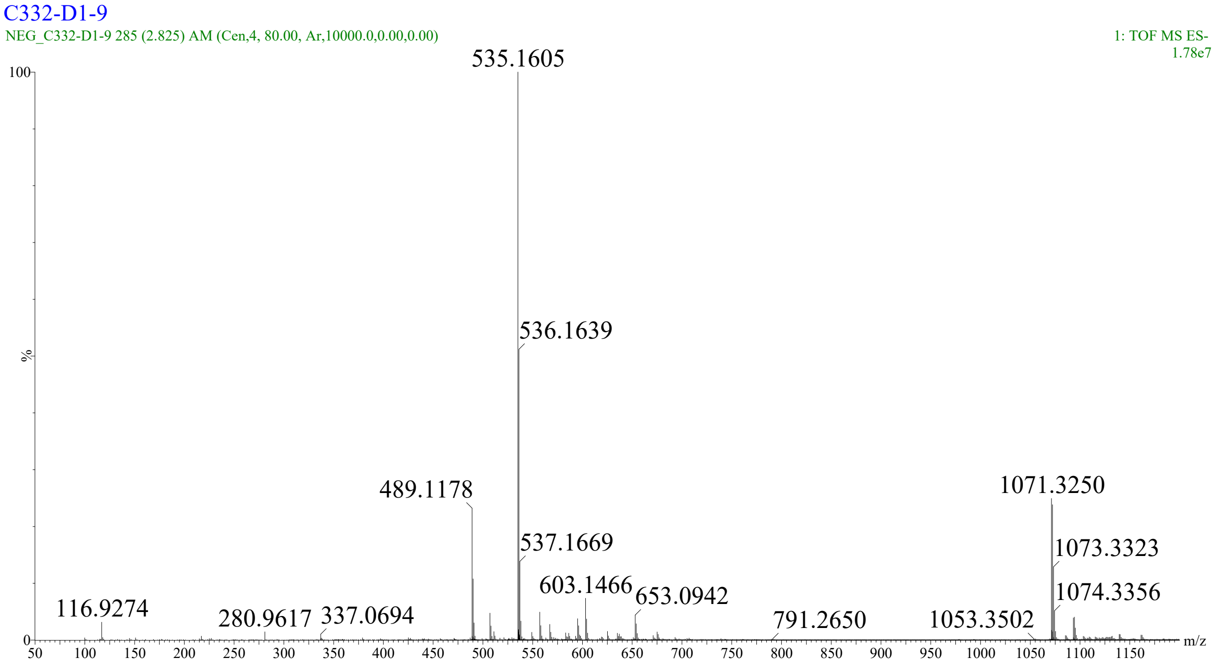

Figure S18. HR-ESI-MS spectrum of compound **3**

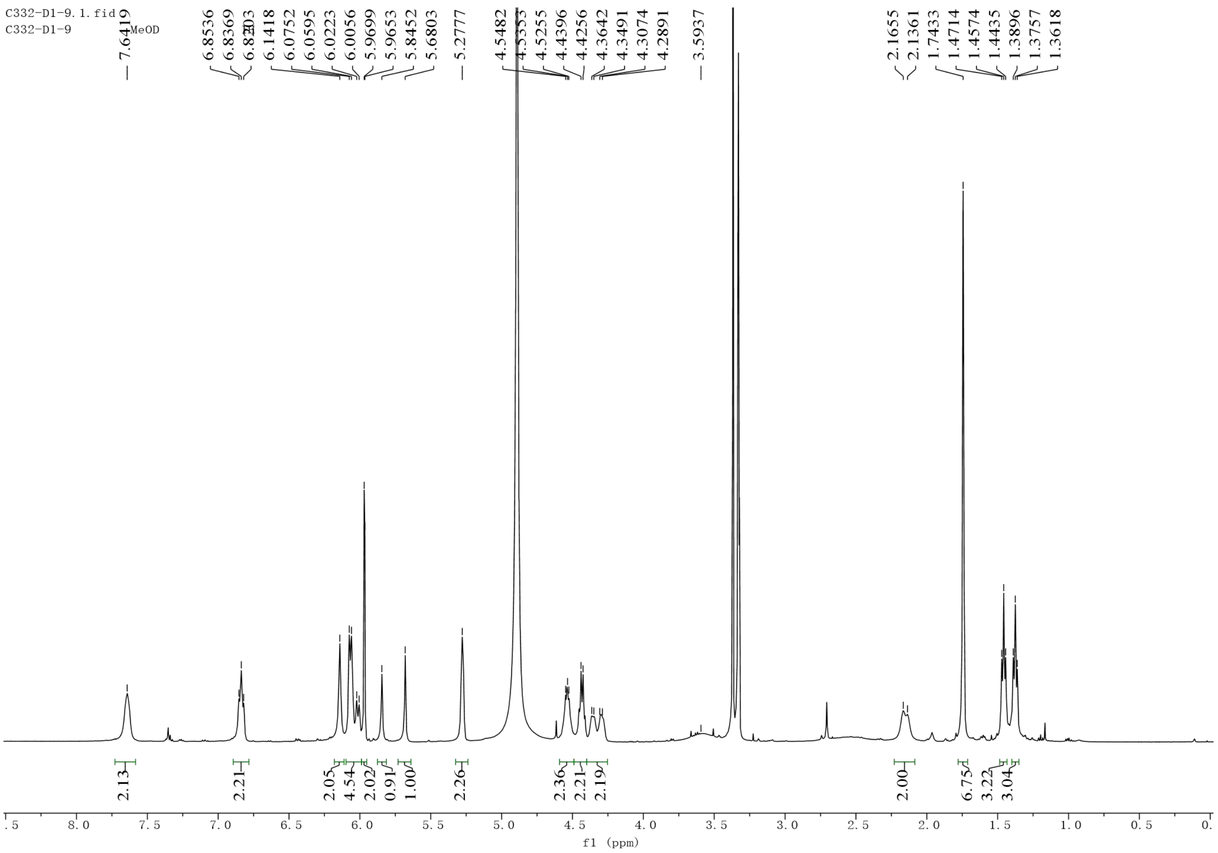

Figure S19. ^{1}H-NMR spectrum of compound **3** in CD_3OD

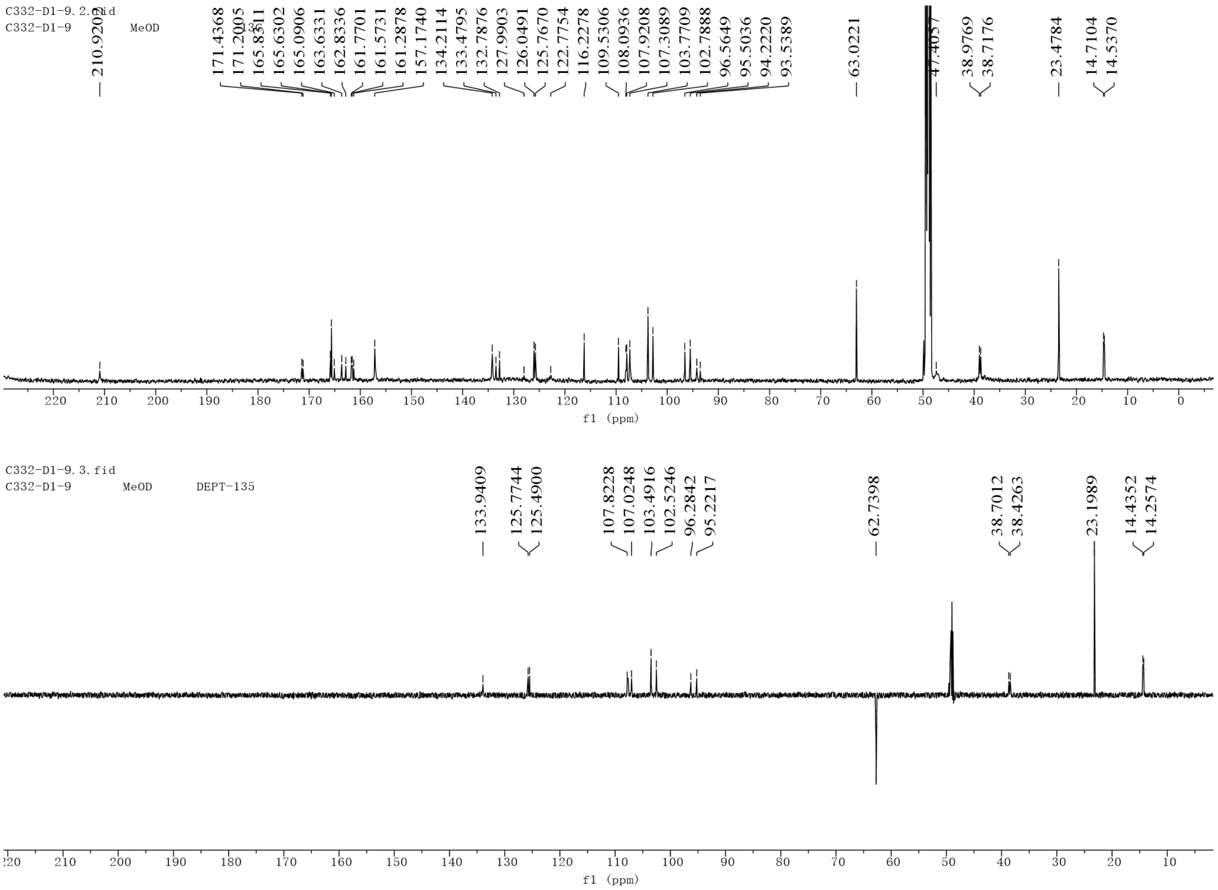

Figure S20. ^{13}C-NMR and DEPT spectra of compound **3** in CD_3OD

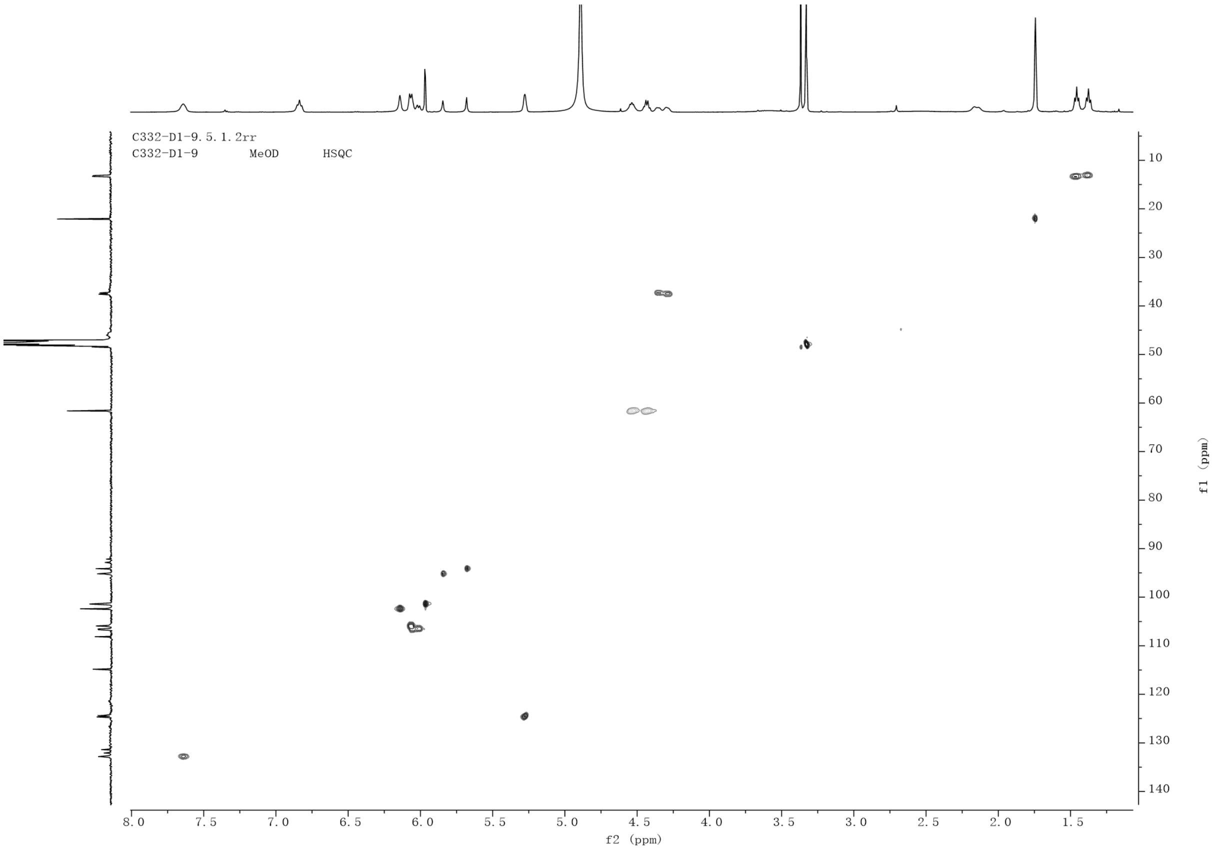

Figure S21. HSQC spectrum of compound **3**

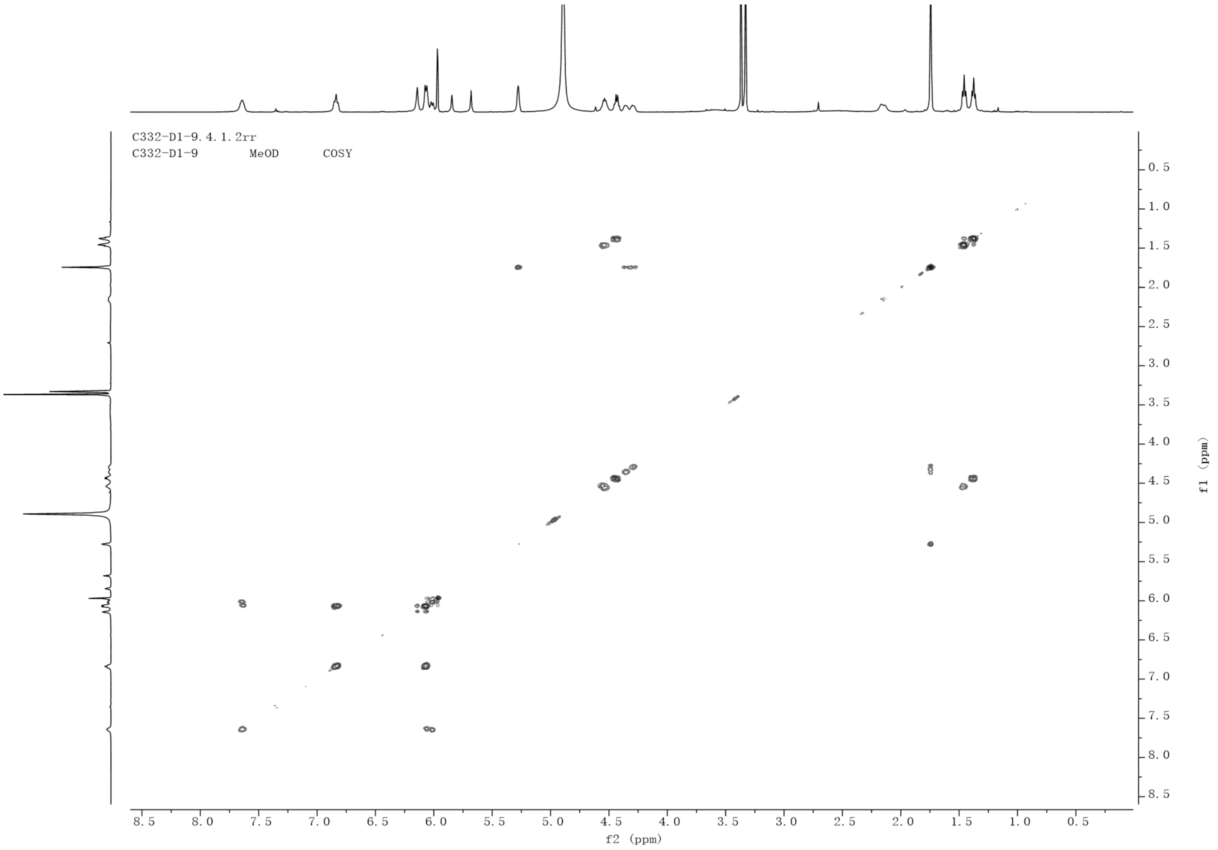

Figure S22. ^{1}H-^{1}H COSY spectrum of compound **3**

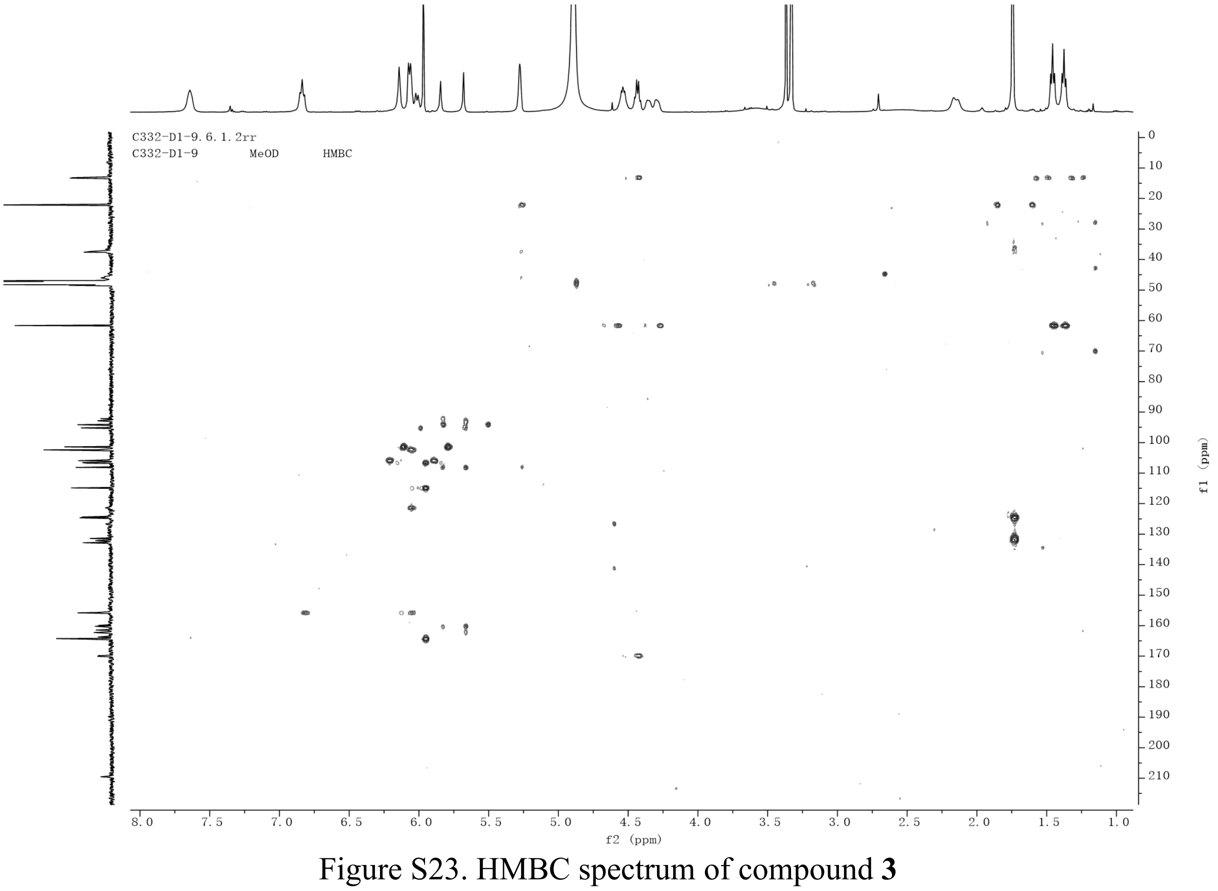

Figure S23. HMBC spectrum of compound **3**

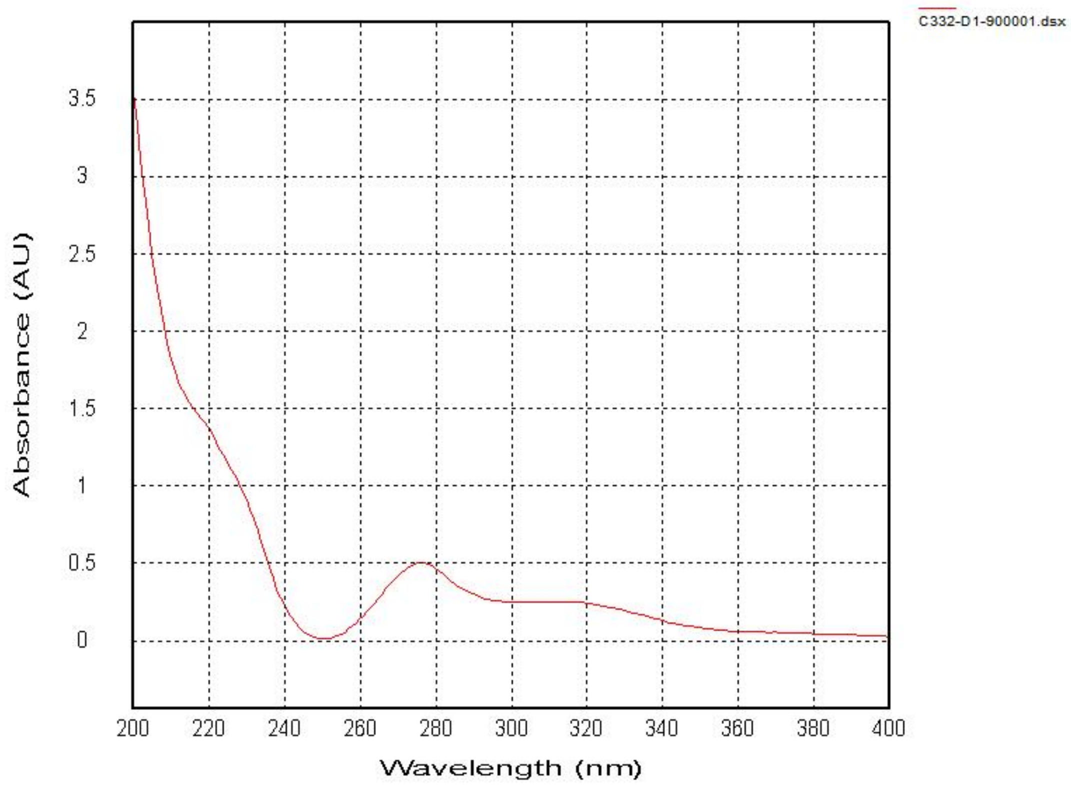

Figure S24. UV spectrum of compound **3**

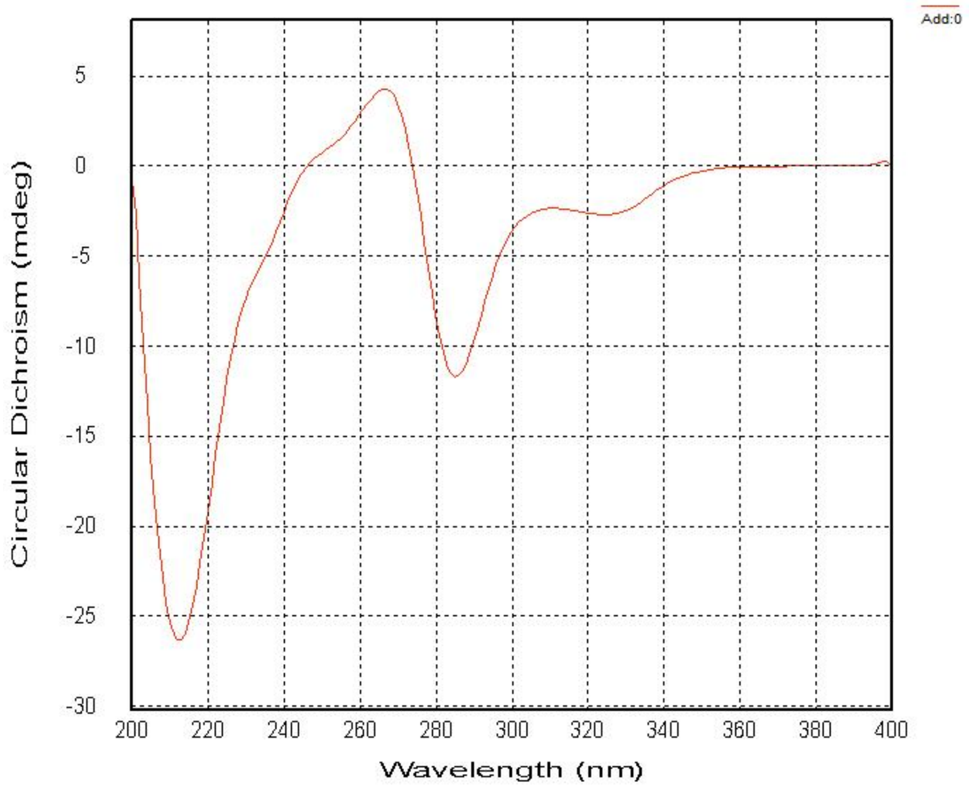

Figure S25. ECD spectrum of compound **3**

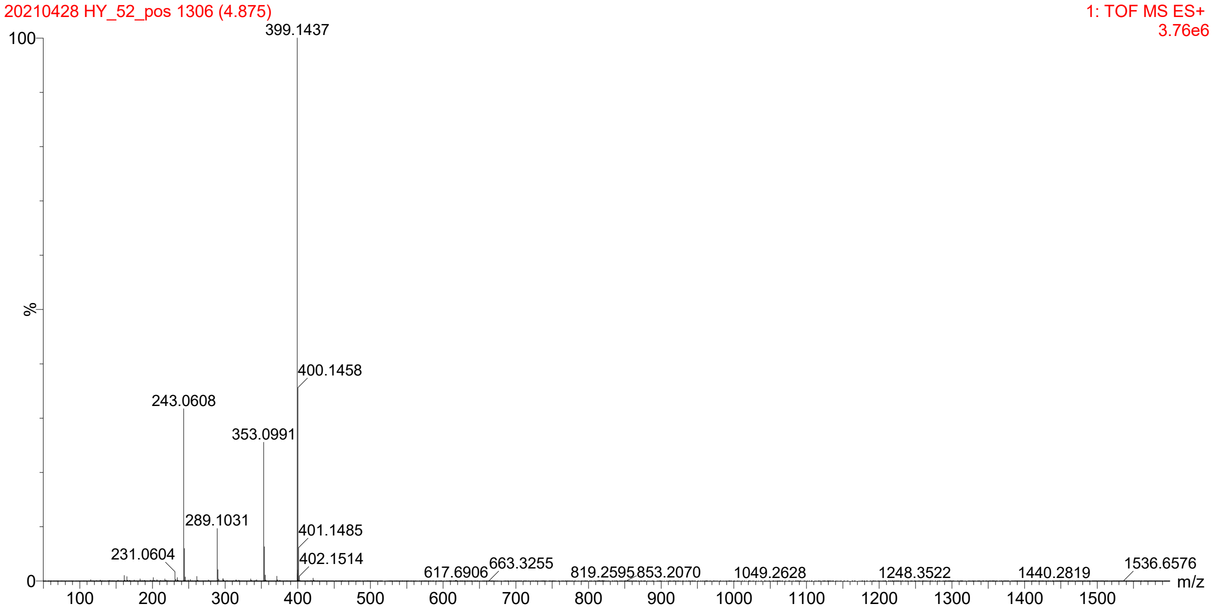

Figure S26. HR-ESI-MS spectrum of compound **4**

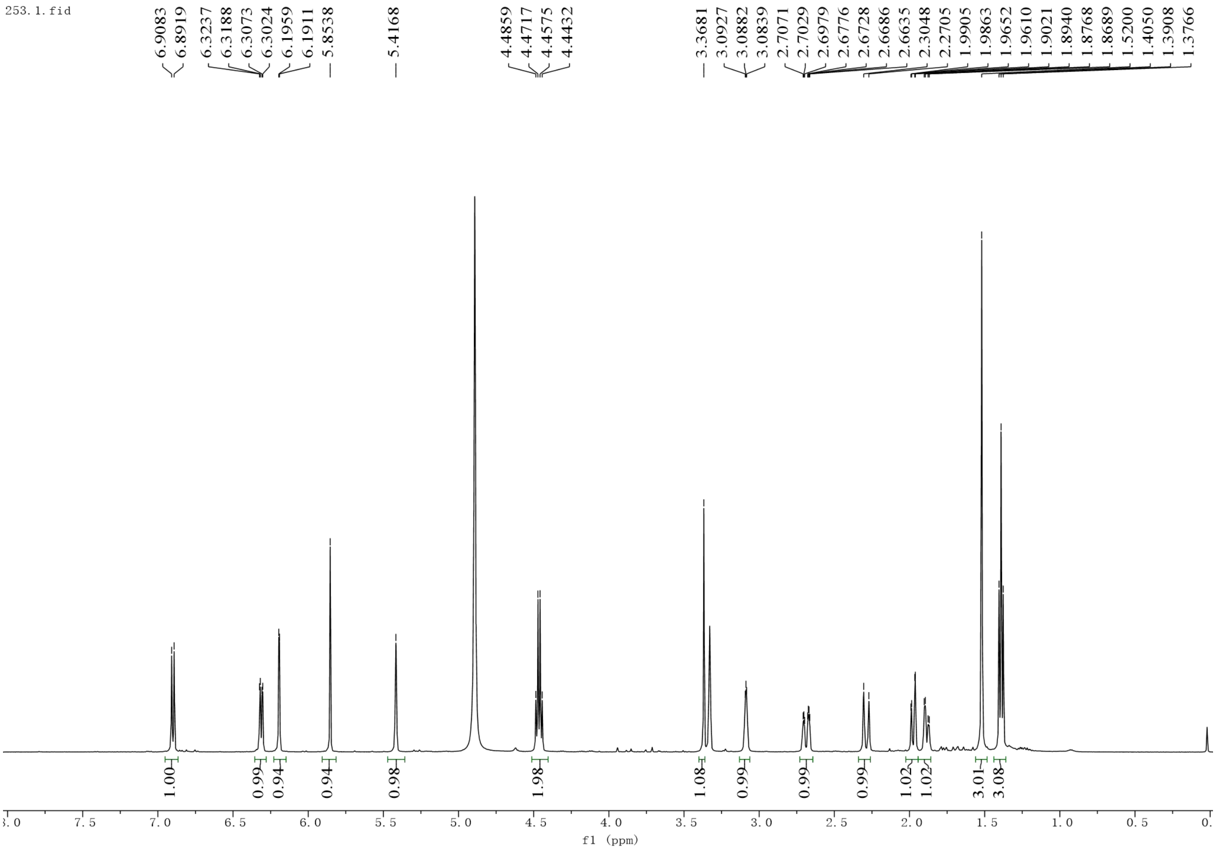

Figure S27. ^{1}H-NMR spectrum of compound **4** in CD_3OD

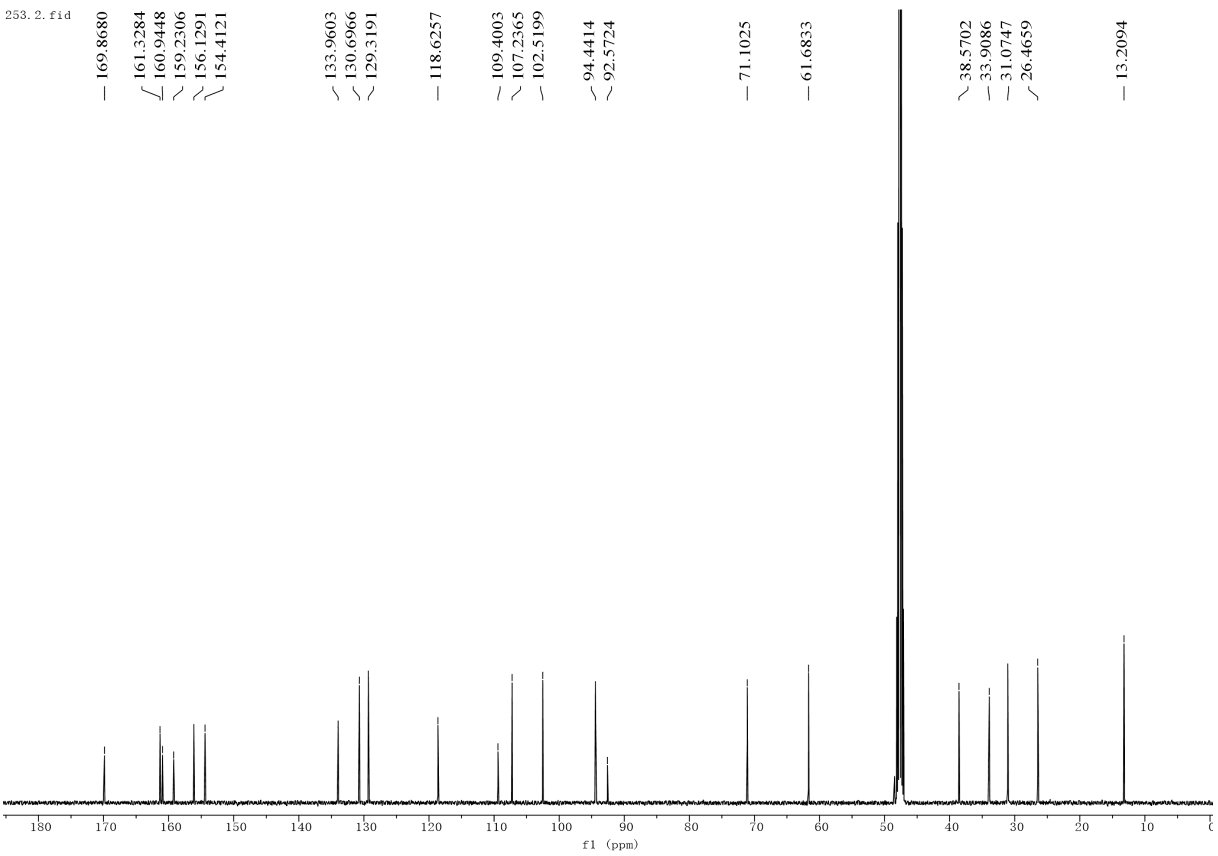

Figure S28. ^{13}C-NMR spectrum of compound **4** in CD_3OD

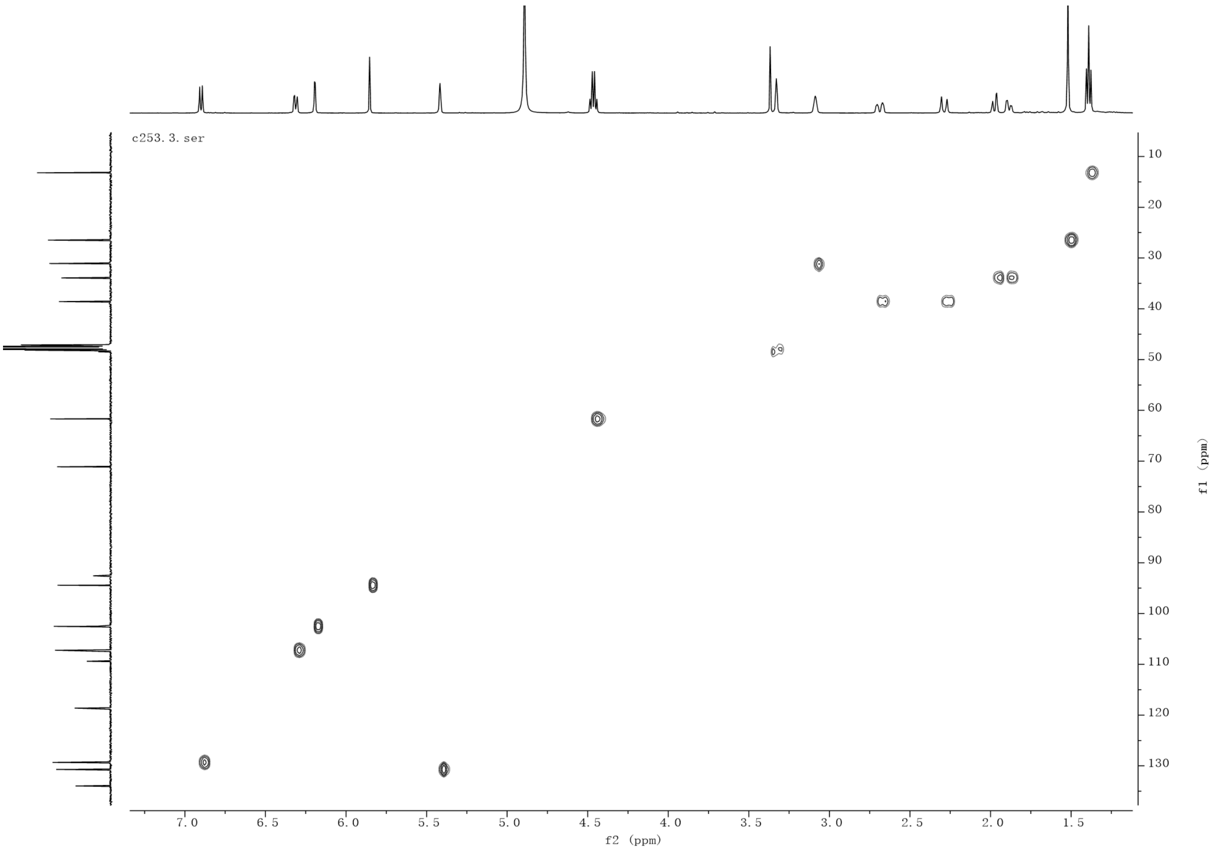

Figure S29. HSQC spectrum of compound **4**

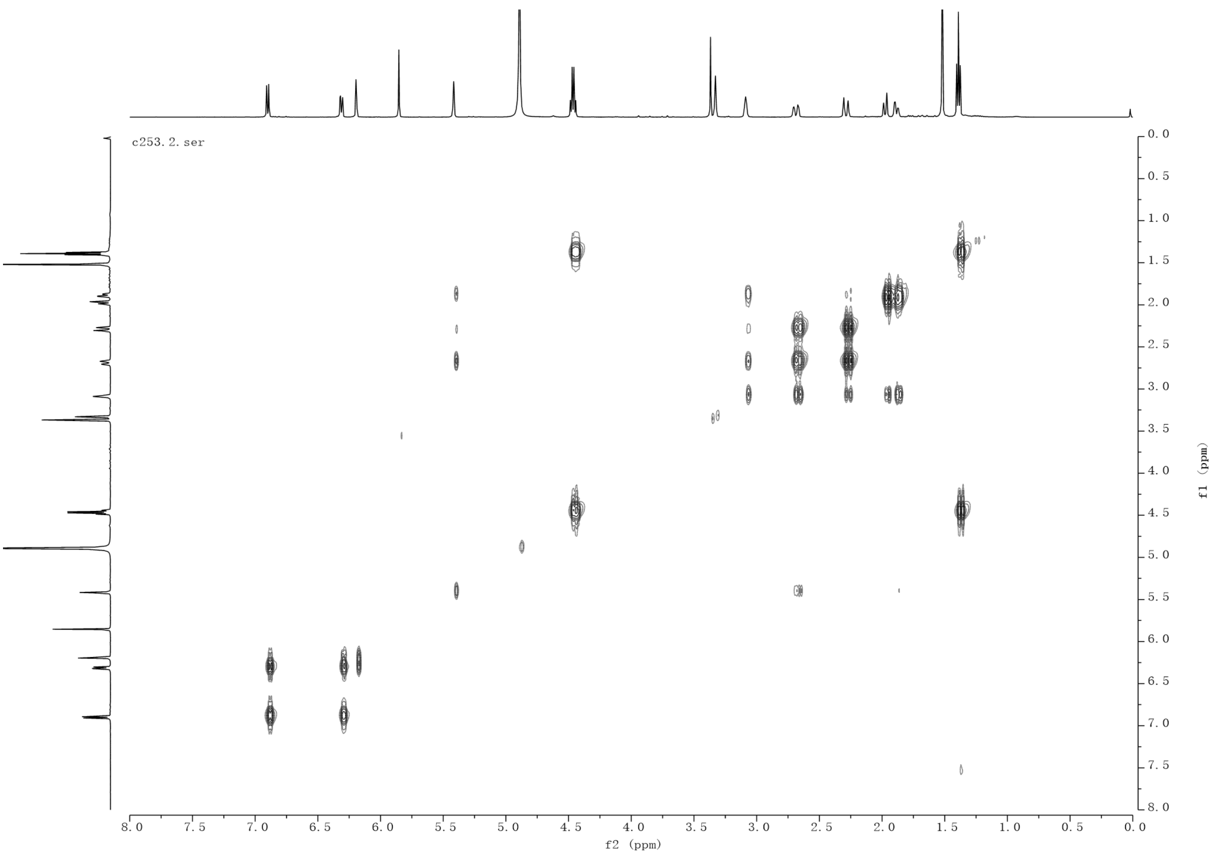

Figure S30. ^{1}H-^{1}H COSY spectrum of compound **4**

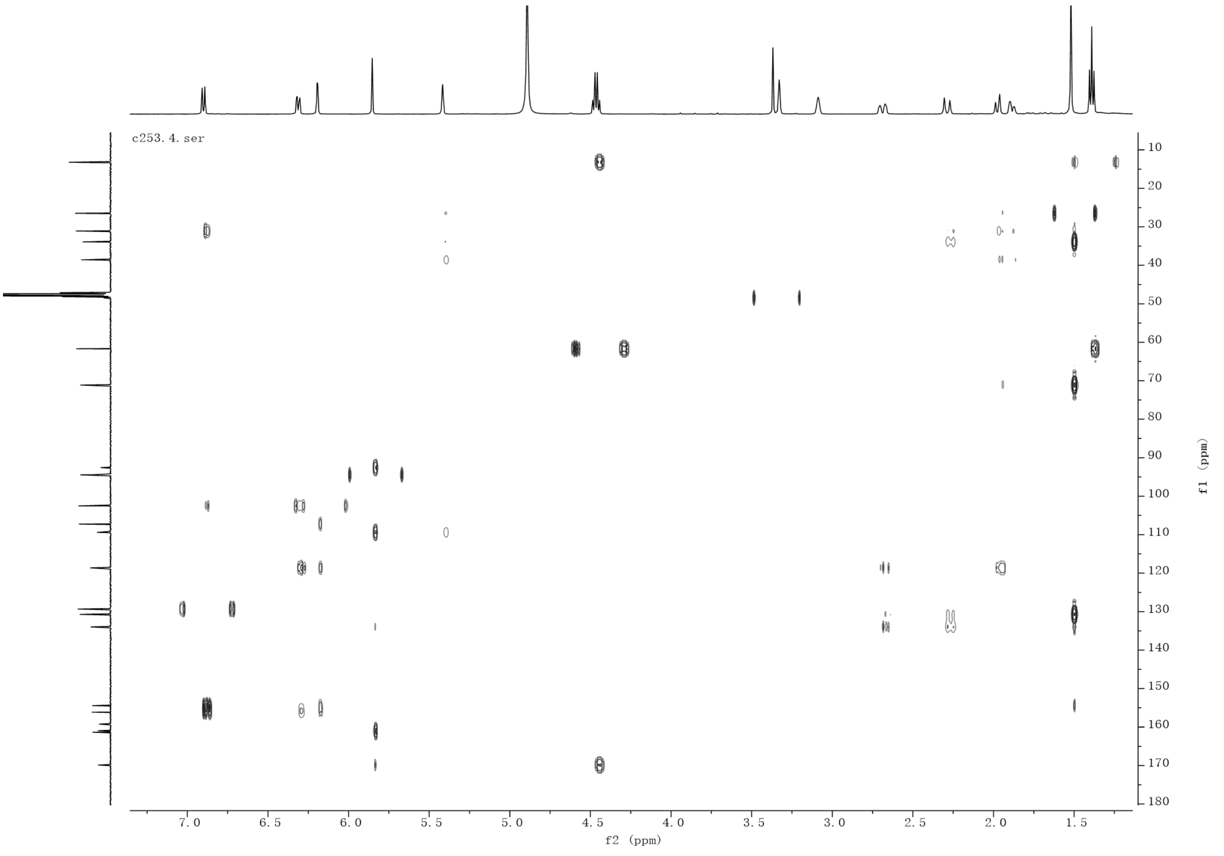

Figure S31. HMBC spectrum of compound **4**

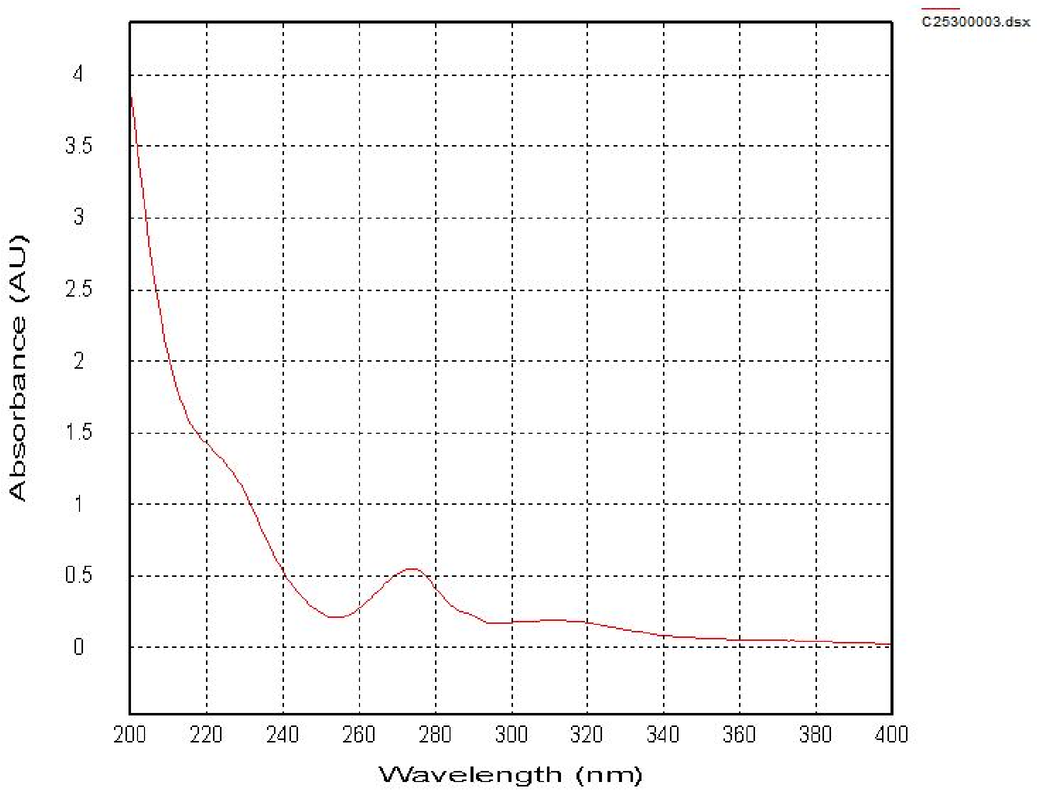

Figure S32. UV spectrum of compound **4**

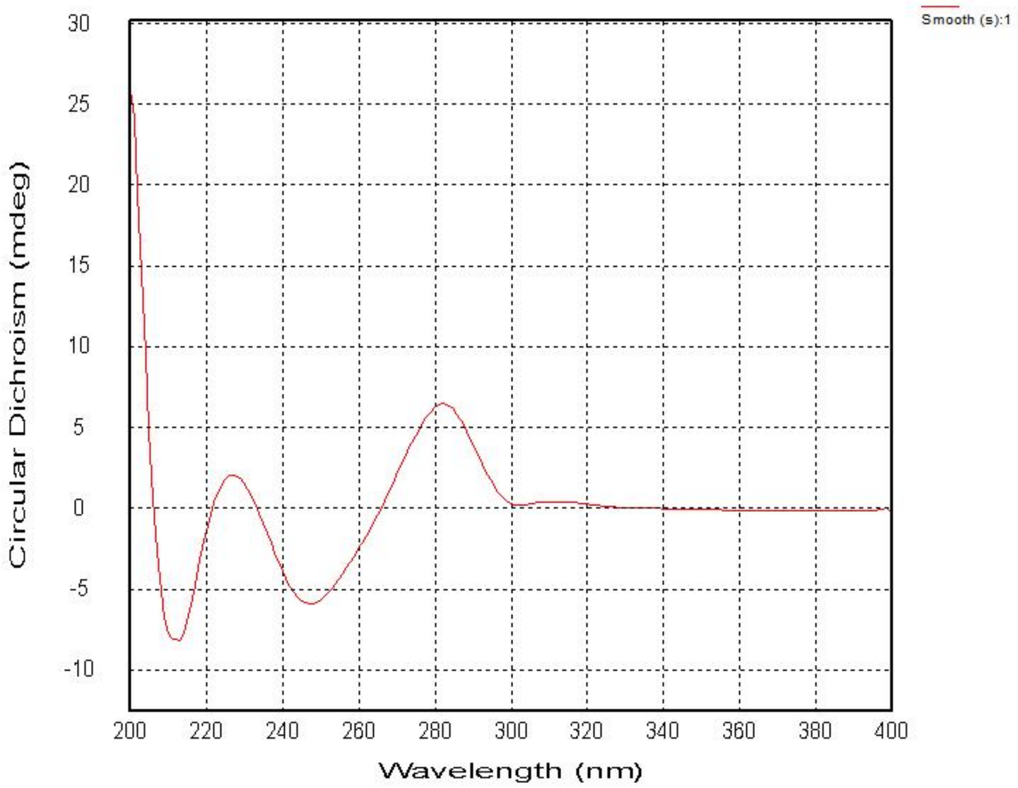

Figure S33. ECD spectrum of compound **4**

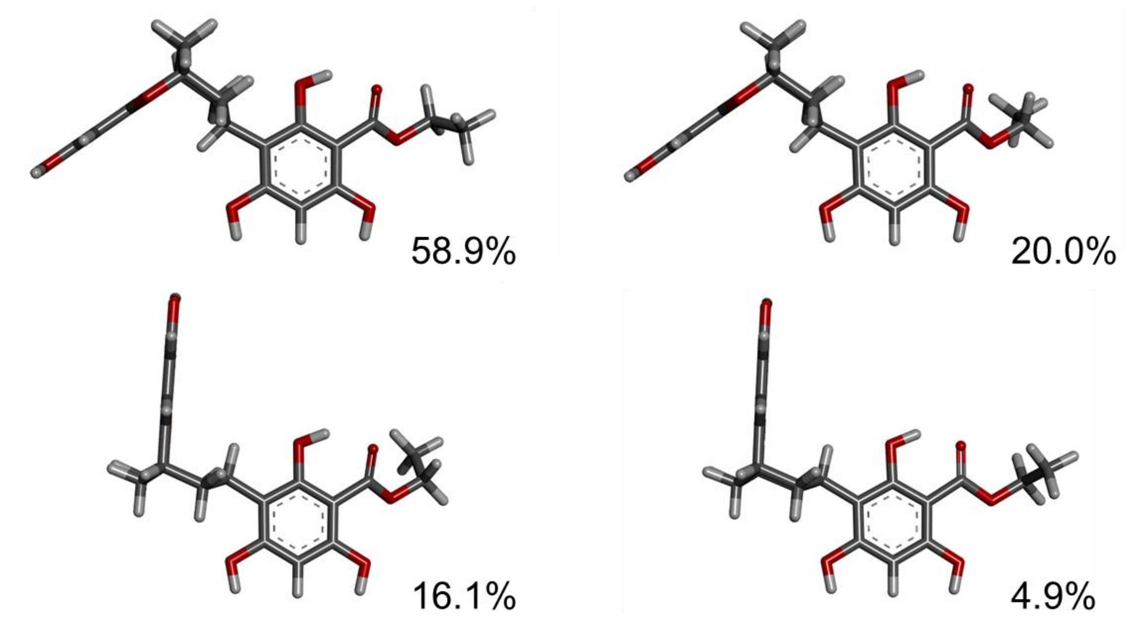

Figure S34. Low-energy conformation of compound **4** optimized by DFT

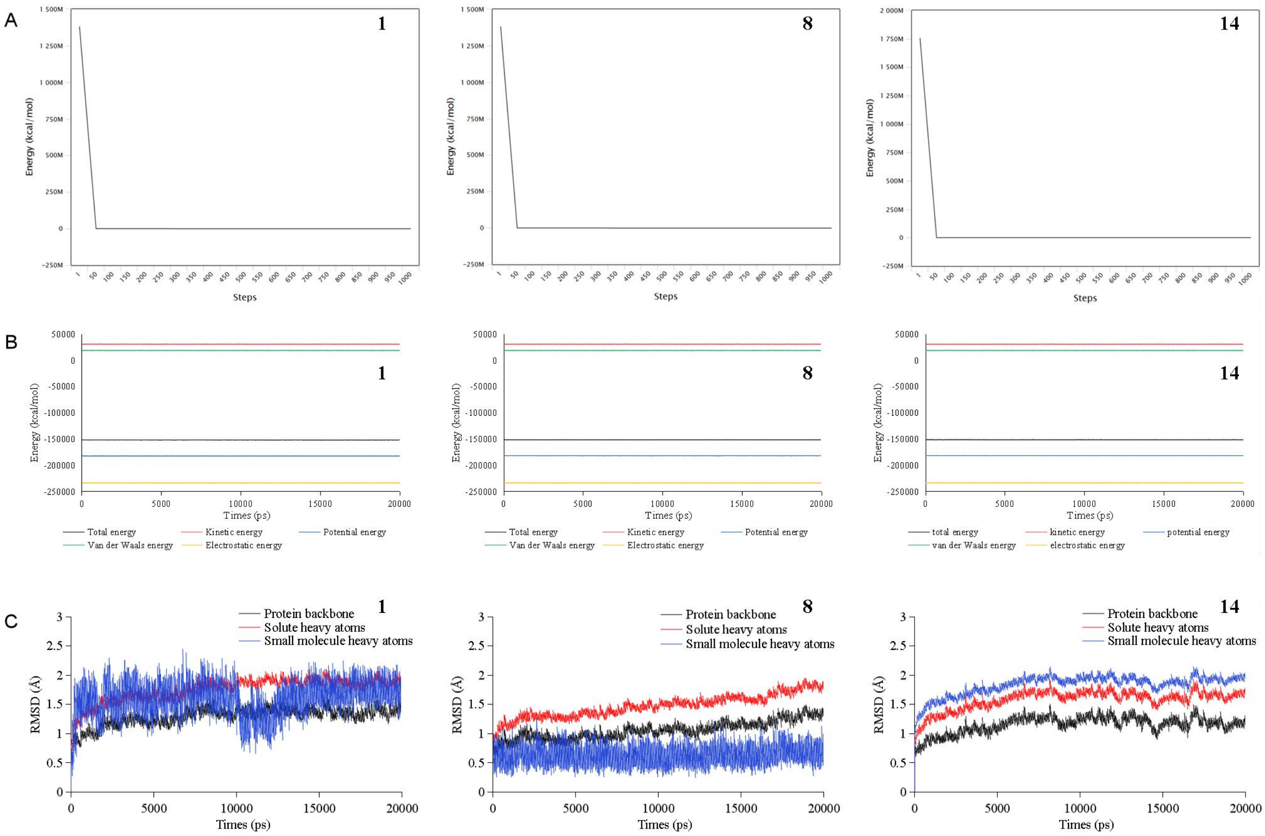

Figure S35. Molecular dynamics simulation stability analyses of the inhibitors-BChE complex.

(A) Potential energy changes during 1000 steps energy minimization of the inhibitors-BChE complex. (B) Time courses of total energy, potential energy, kinetic energy, van der Waals energy, and electrostatic energy for the inhibitors-BChE complex during production simulation. (C) RMSD values of the BChE protein backbone and inhibitors heavy atoms during 20 ns NVT production simulation.
